# Reported Dosing‐Related Medication Errors Involving Systemic Antibiotics in the Brazilian Pharmacovigilance Database (2018–2025): Distribution Across the WHO AWaRe Classification

**DOI:** 10.1002/pds.70496

**Published:** 2026-10-07

**Authors:** Valcieny Sandes, Alice Ramos‐Silva, Adriana Ivama‐Brummell, Albert Figueras, Elisangela Costa Lima

**Affiliations:** ^1^ Postgraduate Programme in Pharmaceutical Sciences, School of Pharmacy, Federal University of Rio de Janeiro Rio de Janeiro/RJ Brazil; ^2^ Laboratory of Pharmacoepidemiology and Clinical Pharmacy, School of Pharmacy, Federal University of Rio de Janeiro Rio de Janeiro/RJ Brazil; ^3^ Pharmacovigilance Office, Post‐Marketing Surveillance General‐Office, Brazilian Health Regulatory Agency (Anvisa) Brasília/DF Brazil; ^4^ Independent Researcher, Pharmacovigilance Consultant Santos/SP Brazil

**Keywords:** administration and dosage, anti‐bacterial agents, drug resistance, medication error, pharmacovigilance

## Abstract

**Purpose:**

Appropriate antibiotic dosing is essential to minimise treatment failure and reduce selective pressure that may contribute to antimicrobial resistance. This study characterised antibiotic medication‐error drug–event pairs (MEPs) reported to the medicines Individual Case Safety Reporting (ICSR) system, VigiMed and assessed the distribution of dosing‐related error drug–event pairs (DEPs) across World Health Organization (WHO) AWaRe (Access, Watch, and Reserve) antibiotic classes.

**Methods:**

We conducted an exploratory cross‐sectional analysis of reports involving systemic antibiotics (Anatomical Therapeutic Chemical, ATC Classification System as J01 ‐ antibacterials for systemic use) from Brazil's open‐access pharmacovigilance database (VigiMed) from 2018 to 2025. MEPs were identified using the Standardised MedDRA Query for Medication Error; within this group, Preferred Terms (PTs) related to dose amount, timing, frequency, duration, or rate of administration were classified as DEPs (32 PTs). Descriptive analyses and modified Poisson regression were used to examine demographic and reporting characteristics associated with MEP classification and, among MEPs, the association between AWaRe class and DEP classification.

**Results:**

The adjusted prevalence of MEP classification was higher among neonates (PR 3.90), infants (PR 1.78) and older adults (PR 1.70) than among adults. DEPs accounted for 31.7% of MEPs (*n* = 2587; 61 antibiotics), with dose omission (51.4%) and dosing regimen problems (16.7%) as the leading error subtypes. Among DEPs, 35.9% involved Access, 56.7% Watch and 7.1% Reserve antibiotics. In adjusted analyses, Watch (PR 1.14; 95% CI 1.07–1.22) and Reserve (PR 1.31; 95% CI 1.16–1.49) antibiotics showed a higher prevalence of DEPs than Access antibiotics.

**Conclusions:**

This study analyses pharmacovigilance data on antibiotic dosing‐related medication errors, highlighting potential opportunities for antimicrobial stewardship.

## Key Points

- One‐third of antibiotic medication‐error drug–event pairs reported in Brazil's national pharmacovigilance system (VigiMed) involved dosing‐related errors, mainly omissions and dosing regimen problems, with potential to compromise treatment effectiveness.
- Drug–event pairs involving neonates, infants and older adults showed a higher proportion of medication‐error classification than those involving adults.
- Reported dosing‐related error drug–event pairs involving Watch and Reserve antibiotics highlight potential vulnerabilities in the use of antibiotic classes prioritised for antimicrobial stewardship interventions.
- The characterisation of inappropriate antibiotic dosing in Brazil's national pharmacovigilance database provides insight into patterns of suboptimal medicine use with potential implications for antimicrobial resistance and may support targeted stewardship actions.
- Although spontaneous reporting systems do not permit estimation of population‐level risk, they represent an underexplored data source and can complement other surveillance approaches to inform antimicrobial stewardship.

## Plain Language Summary

Antibiotics need to be given in the right amount and at the right time to work well, avoid treatment failure and help reduce antibiotic resistance. We studied reports sent to Brazil's medicines Individual Case Safety Reporting (ICSR) system, VigiMed, between 2018 and 2025. A single report can include several antibiotics and several adverse events, creating multiple drug–event pairs. We looked for dosing problems involving the amount given, missed doses, how often the medicine was given, how long treatment lasted and how quickly it was administered. Drug–event pairs involving newborns, infants and older adults were more likely to be classified as medication errors than those involving adults. Dosing‐related drug–event pairs made up 31.7% of all medication‐error drug–event pairs, with 2587 pairs involving 61 antibiotics. Missed doses were the most common problem (51%), followed by problems with the dosing schedule (17%). The World Health Organization (WHO) groups antibiotics into Access, Watch and Reserve (AWaRe) categories to guide appropriate use. Access antibiotics are generally preferred for common infections, while Watch antibiotics require closer monitoring and Reserve antibiotics are usually kept for infections where other treatment options are limited. Among dosing‐related drug–event pairs, 35.9% involved Access antibiotics, 56.7% Watch antibiotics and 7.1% Reserve antibiotics. Within the medication‐error group, the proportion classified as dosing‐related was higher for Watch and Reserve antibiotics than for Access antibiotics. Because VigiMed does not include every antibiotic treatment given in Brazil, the study cannot show how common these errors are in all patients. However, the findings may help health services identify where safer dosing and closer monitoring are most needed.

## 1 Introduction

Antimicrobial resistance (AMR) remains a major global health challenge. Beyond the choice of antibiotic, correct dosing and timely administration are critical to ensure adequate drug exposure and clinical effectiveness [1, 2, 3, 4, 5, 6].

Medication errors involving antibiotics, particularly dose omissions and dosing schedule deviations, have been repeatedly documented in hospital settings [7, 8, 9]. Such errors may compromise treatment effectiveness and are of particular concern in vulnerable populations such as neonates and older adults [10, 11]. Inadequate or interrupted antibiotic exposure may also contribute to AMR [6, 12, 13].

Pharmacovigilance refers to the systematic monitoring and evaluation of medicine‐related safety problems after use in real‐world settings [14, 15]. This scope, which extends beyond adverse drug reactions, is particularly relevant to medication errors, which represent potentially preventable problems in the medication‐use process and may provide important signals for patient safety surveillance [16, 17, 18].

In Brazil, VigiMed, the Brazilian Individual Case Safety Reporting (ICSR) system, provides open‐access national pharmacovigilance data that can be used to characterise such medication‐use problems. However, analyses focusing specifically on antibiotic dosing‐related errors remain limited, and evidence from Latin America is scarce [19, 20, 21, 22]. Moreover, the distribution of such errors across antimicrobial stewardship–relevant categories, such as the World Health Organization (WHO) AWaRe (Access, Watch, and Reserve) classification, has not been comprehensively assessed [23, 24].

This study aimed to characterise antibiotic medication‐error drug–event pairs (MEPs) reported to Brazil's national pharmacovigilance system (VigiMed) and to assess whether the proportion classified as dosing‐related differed across WHO AWaRe antibiotic classes.

## 2 Methods

### 2.1 Study Design and Setting

This exploratory cross‐sectional study used secondary data on antibiotic‐related adverse events (AEs) recorded in VigiMed between December 2018, when the system was implemented and November 2025. VigiMed (internationally referred to as VigiFlow) is managed by the Brazilian Health Regulatory Agency (Anvisa), standardised according to ICH E2B (R3) and integrated into the WHO global pharmacovigilance database (VigiBase) [25].

AE reports can be submitted spontaneously by individual citizens, healthcare professionals and non‐healthcare professionals, while healthcare services [26] and marketing authorisation holders [27] are required to report. A single report may include multiple medicines and AEs. Events were analysed as reported, without causality assessment.

Individual Case Safety Reports (ICSRs) involving antibiotics classified according to the Anatomical Therapeutic Chemical (ATC) Classification System as J01 (antibacterials for systemic use) [28] were retrieved from the Brazilian Government Open Data Portal [29] (reports, medicines and reactions datasets) on 3 December 2025. Active substances were coded according to the WHODrug Global Dictionary [30]. AEs were coded using the Medical Dictionary for Regulatory Activities (MedDRA) [31]. The analytical unit was the drug–event pair.

### 2.2 Data Processing

A hierarchical deterministic deduplication strategy was applied to identify duplicate reports (see Supplementary Methods S1 and Table S1 for the detailed algorithm) [32, 33, 34]. Composite keys were used at two sequential levels, prioritising complete records and minimising false‐positive merges. Within duplicate groups, one record was retained and group identifiers were preserved for auditability. Eligible reports were required to contain at least one antibiotic and one AE. Reports involving foetuses were excluded from the analysis.

Age and active substance variables were harmonised to ensure consistency and comparability across reports, as detailed in Supplementary Methods S2 and Table S2 [35].

A report refers to the ICSR submitted to VigiMed, whereas a drug–event pair refers to each combination of one medicine and one reported AE. Because a single report may include multiple antibiotics and AEs, reports were expanded to generate all possible drug–event pairs and analyses were conducted at this level.

### 2.3 Analytical Selection

All eligible drug–event pairs involving systemic antibiotics (ATC J01) reported to VigiMed as ‘suspect’ or ‘medicine not administered’ during the study period were included. Drug–event relationship coding was taken from the medicine‐level dataset, where the relationship is recorded separately for each medicine.

MEPs were identified using the Standardised MedDRA Query (SMQ) for Medication Error, Brazilian Portuguese version 28.1, which comprised 221 Preferred Terms (PTs). For MedDRA term selection and analysis, a medication error was defined as any unintentional and preventable event that may cause or lead to inappropriate medication use or patient harm while the medication is under the control of a healthcare professional, patient, or consumer [36, 37].

Dosing‐related error drug–event pairs (DEPs) were defined as a study‐specific subset of MEPs with PTs explicitly related to dose amount, dose omission, dose adjustment or titration, dosing frequency or interval, duration of administration, or rate of administration; 32 PTs were selected to form this subset (Supplementary Methods Table 1 presents the terms identified in the study dataset and their corresponding classification).

Antibiotics were categorised as Access, Watch, Reserve, Not classified or Not recommended using the 2023 WHO AWaRe classification, which considers their potential impact on AMR and stewardship priorities [24].

Further details are provided in Supplementary Methods S3 [38].

### 2.4 Data Analysis and Statistics

Descriptive analyses characterised the antibiotics involved, age group, sex, reporting source and event seriousness. DEPs were examined separately and grouped into two broad domains according to the primary aspect of dosing affected by each PT. Timing‐related errors included dose omission and dosing regimen problems, as these terms mainly refer to whether a scheduled dose was administered, adjusted, or titrated appropriately and to the frequency, interval or duration of administration. Dosage‐related errors included incorrect dose, subtherapeutic dose and overdose, as these terms primarily refer to the amount of antibiotic administered or prescribed and the resulting level of drug exposure.

We described the distribution of drug–event pairs by year and AWaRe class. A heatmap was generated to show the proportion of DEPs among MEPs by individual antibiotic and age group, excluding antibiotics with sparse data (< 5 MEPs). The heatmap was descriptive and did not represent adjusted associations.

Modified Poisson regression with a log link was used to estimate prevalence ratios (PRs) and 95% confidence intervals (CIs) for the binary outcomes [39, 40, 41]. Cluster‐robust variance at the report level was used to account for multiple drug–event pairs originating from the same report. Crude PRs were estimated using univariable models, and adjusted PRs were estimated using multi‐variable models. Each PR compared the proportion of drug–event pairs meeting the specified binary outcome in a given category with the corresponding proportion in the reference category, within the respective analytical population.

As a contextual analysis, we examined demographic and reporting characteristics associated with medication‐error classification among all eligible drug–event pairs by comparing pairs with and without medication errors. The multi‐variable model included age group, sex and reporting source.

The main regression analysis examined whether the proportion of DEPs differed by AWaRe class among antibiotic MEPs. AWaRe class was the exposure, with Access as the reference category and Watch and Reserve as comparison categories, and dosing‐related error classification was the binary outcome. The multivariable model included age group, sex and reporting source. Antibiotics classified as ‘Not classified’ or ‘Not recommended’ were excluded because of their low frequency (< 1% of MEPs).

Reporting‐source categories containing fewer than 10 drug–event pairs in the respective analytical population were excluded from the regression analyses because of sparse data and unstable parameter estimation. Event seriousness was not included in either regression model because it could be a consequence of the medication error rather than a determinant, and its inclusion could introduce bias.

The main analysis was repeated in two sensitivity analyses: (i) after restricting the sample to drug–event pairs with a ‘suspect’ drug–event relationship and (ii) after adjustment for calendar year of entry into the system. Calendar year was included as a categorical covariate. During the study period, the pharmacovigilance system was implemented and became established while the COVID‐19 pandemic was occurring. Because these temporal effects could not be separated analytically, calendar year was treated only as an aggregate temporal adjustment. We used year of entry because it was available for all records, whereas reporting year was available for only 51.2% of records.

Because spontaneous reporting systems lack population denominators and are subject to reporting biases, the regression analyses were used to explore patterns of association among reported drug–event pairs rather than to estimate incidence or population‐level risks. Missing data were not imputed because missingness was unlikely to be random. Missing values were retained as a distinct ‘Not reported/Unknown’ category in the descriptive and regression analyses and were considered when interpreting the results.

This cross‐sectional study was reported in accordance with the Strengthening the Reporting of Observational Studies in Epidemiology (STROBE) statement [42]; the completed checklist is provided in Table S9.

Statistical significance was defined as a two‐sided *p*‐value < 0.05. Analyses were performed using Python (version 3.13).

ChatGPT (OpenAI) was used to draft and refine scripts for data cleaning, deduplication, construction of antibiotic drug–event pairs and statistical analyses. All code was reviewed, modified and verified by the authors, who take full responsibility for the accuracy and integrity of the analyses.

## 3 Results

The analysis comprised 63 198 eligible systemic antibiotic drug–event pairs, of which 8170 were classified as MEPs, including 2587 (31.7%) DEPs involving 61 antibiotics (Figure 1). Reports contributing to MEPs had a median of 1.0 antibiotic per report (mean ± SD: 1.05 ± 0.24; range 1–5) and 1.0 PT per report (mean ± standard deviation (SD): 1.18 ± 0.43; range 1–8).

**FIGURE 1:**
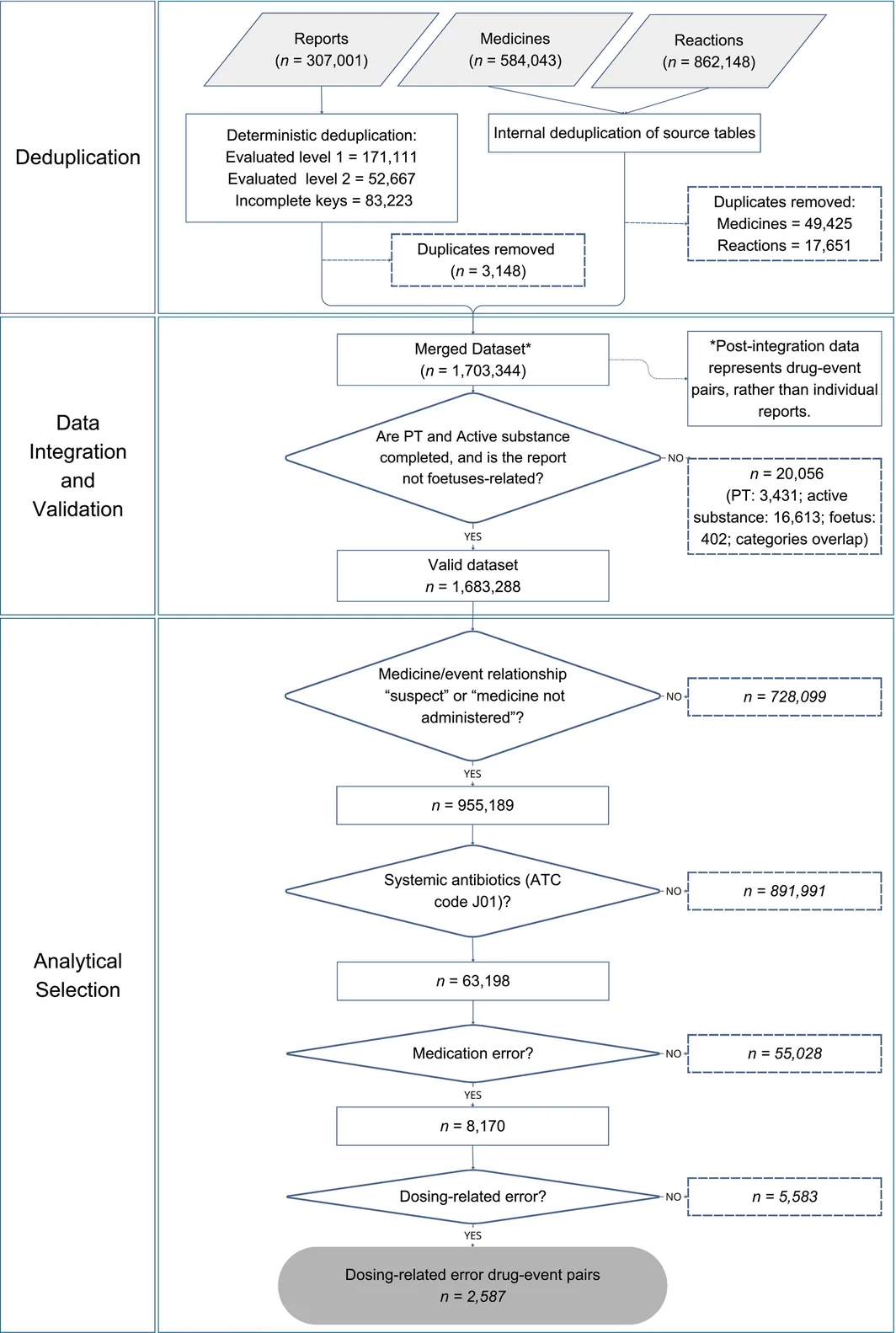
Study flow diagram of data processing and selection. ATC, anatomical therapeutic chemical classification.

Table S1 summarises characteristics of J01 drug–event pairs by error classification. Figure S1 shows the five most frequently reported antibiotics associated with DEPs within each age group, and Table S4 presents frequencies of antibiotics associated with DEPs.

Timing‐related errors accounted for 68.1% of DEPs, comprising dose omission (51.4%) and dosing regimen problems (16.7%). Dosage‐related errors accounted for the remaining 31.9%, comprising incorrect dose (16.2%), subtherapeutic dose (5.5%) and overdose (10.2%) (Table 1).

**TABLE 1:** Distribution of dosing‐related error drug–event pairs by error category in VigiMed (Brazil, 2018–2025).

Error type	‘Medicine not administered’ drug–event pairs	‘Suspect’ drug–event pairs	Total drug–event pairs
Timing			
Dose omission	535 (67.2)	795 (44.4)	1330 (51.4)
Dosing regimen problem	35 (4.4)	397 (22.2)	432 (16.7)
Dosage			
Incorrect dose	50 (6.3)	369 (20.6)	419 (16.2)
Subtherapeutic dose	61 (7.7)	80 (4.5)	141 (5.5)
Overdose	115 (14.4)	150 (8.4)	265 (10.2)
Total	796 (100.0)	1791 (100.0)	2587 (100.0)

*Note:* Data are presented as *n* (%). Percentages were calculated within columns.

MEPs were compared with pairs involving other PTs (Figure 2). Compared with adults, the adjusted prevalence of MEPs was higher among neonates (adjusted PR 3.90; 95% CI 3.45–4.41), infants (adjusted PR 1.78; 95% CI 1.61–1.97) and older adults (adjusted PR 1.70; 95% CI 1.59–1.82). Pairs with unreported age also had a higher adjusted prevalence of MEPs (adjusted PR 4.24; 95% CI 3.97–4.52).

**FIGURE 2:**
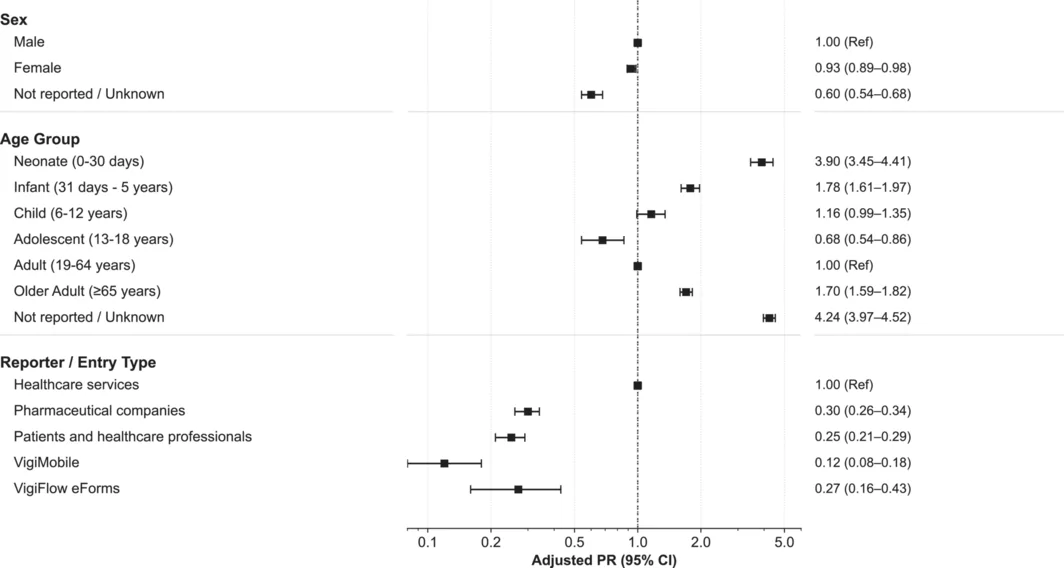
Adjusted prevalence ratios (PRs) and 95% confidence intervals (CIs) for demographic and reporting characteristics associated with medication‐error classification among systemic antibiotic drug–event pairs (ATC J01) reported to VigiMed, (Brazil, 2018–2025). After exclusion of reporting‐source categories containing fewer than 10 drug–event pairs, the regression analysis included 8169 medication‐error pairs and 55 016 pairs without medication errors. Estimates were obtained using a multi‐variable modified Poisson regression model adjusted for age group, sex and reporting source, with cluster‐robust variance at the report level. ATC, anatomical therapeutic chemical classification.

All other reporting‐source categories had a lower adjusted prevalence of medication‐error classification than the healthcare services category. Pairs involving females had a slightly lower adjusted prevalence than those involving males (adjusted PR 0.93; 95% CI 0.89–0.98). Crude and adjusted PRs were generally similar across age groups and reporting sources (Table S5).

Access, Watch and Reserve antibiotics accounted for 39.6% (*n* = 3239), 54.3% (*n* = 4435) and 5.6% (*n* = 461) of MEPs, respectively. The corresponding proportions among DEPs were 35.9% (*n* = 930), 56.7% (*n* = 1466) and 7.1% (*n* = 183). Antibiotics not classified in AWaRe accounted for 27 pairs (0.3%), six of which were DEPs, whereas not‐recommended antibiotics accounted for eight pairs (0.1%), including two DEPs.

Figure S2 shows the distribution of drug–event pairs by year and AWaRe class. The number of pairs increased over the study period, whereas the distribution across AWaRe classes remained broadly stable.

The proportion of DEPs varied across antibiotics and age groups (Figure 3). Patterns were more readily interpretable in strata with larger numbers of drug–event pairs. Among Watch antibiotics, this was particularly the case for ceftriaxone, piperacillin/tazobactam, vancomycin and meropenem, especially in the adult and older‐adult strata.

**FIGURE 3:**
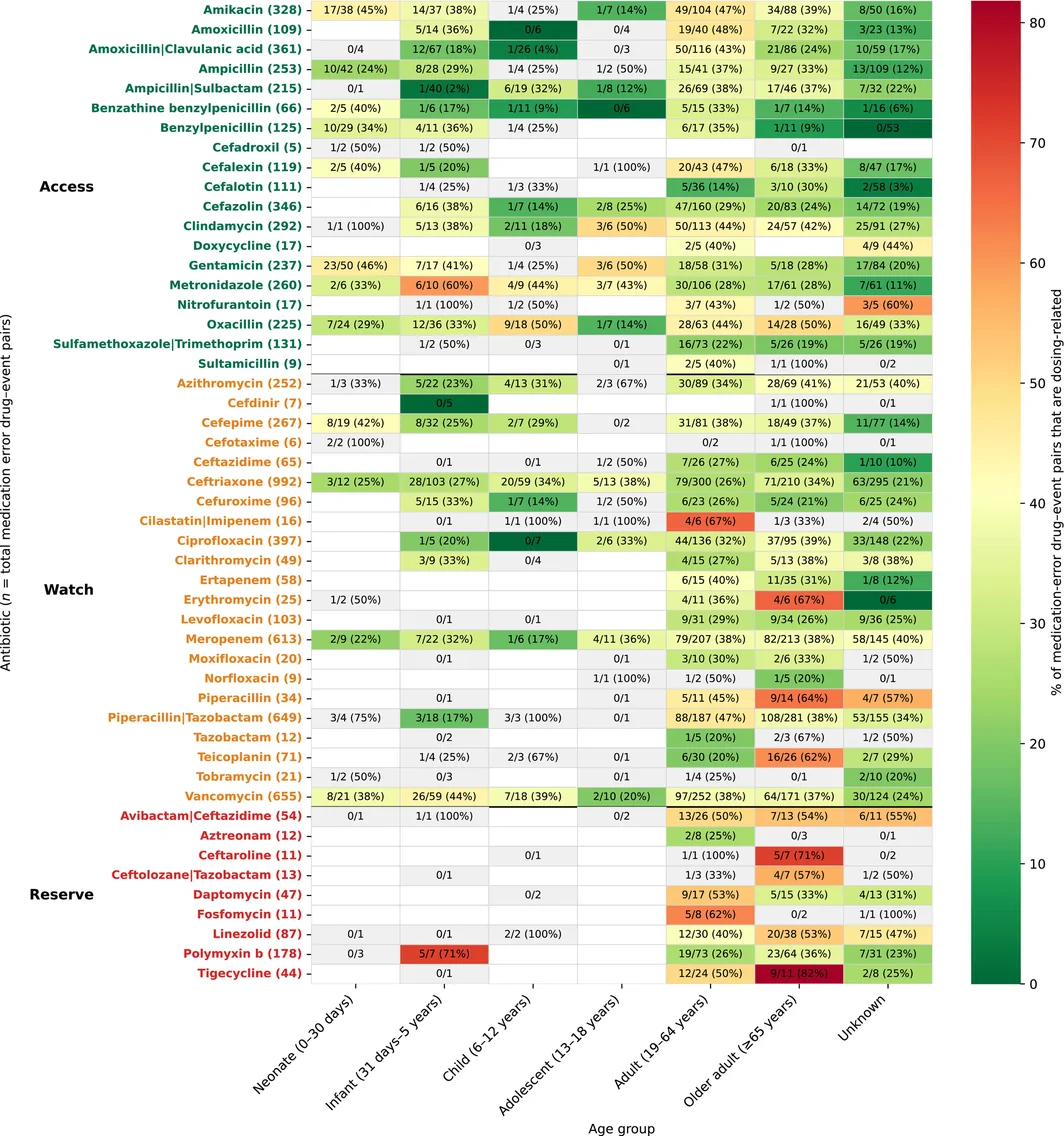
Proportion of dosing‐related error drug–event pairs among antibiotic medication‐error drug–event pairs, by age group and AWaRe classification, VigiMed (Brazil, 2018–2025). Each row represents an antibiotic classified according to the WHO AWaRe grouping. The number in parentheses next to the antibiotic name (*n*) indicates the total number of medication‐error drug–event pairs for that antibiotic included in the analysis. Antibiotics were included if they had at least five medication‐error drug–event pairs overall. Within each cell, the first value shows the number of dosing‐related error drug–event pairs out of the total medication‐error drug–event pairs in that stratum (antibiotic × age group; e.g., 3/12) and the percentage in parentheses is the proportion that were dosing‐related. Cell colour encodes this proportion and is shown only for strata with at least five drug–event pairs; strata with one to four pairs are displayed in grey, because the proportion is unstable, although their values are still shown; strata with no pairs are left blank. Cells with small numbers of drug–event pairs should be interpreted cautiously.

In the adjusted analysis, Watch and Reserve antibiotics had a higher prevalence of DEPs than Access antibiotics (Table 2). Estimates for covariates included in the adjusted model (age group, sex and reporting source) are presented in Table S6.

**TABLE 2:** Association between AWaRe class and dosing‐related error classification among antibiotic medication‐error drug–event pairs, VigiMed (Brazil, 2018–2025).

Variable	Category	PR (95% CI)	*p*
AWaRe class	Access	Reference	
	Watch	1.14 (1.07–1.22)	< 0.001
	Reserve	1.31 (1.16–1.49)	< 0.001

*Note:* Adjusted prevalence ratios (PRs) and 95% confidence intervals (CIs) were estimated using modified Poisson regression with a log link and cluster‐robust variance at the report level. Models were adjusted for age group, sex and reporting source.

In a sensitivity analysis restricted to drug–event pairs with a ‘suspect’ drug–event relationship (*n =* 5927; Table S7), the association persisted with attenuated magnitude (Watch PR 1.10, 95% CI 1.01–1.19; Reserve PR 1.23, 95% CI 1.05–1.44). After adjustment for calendar year, the association remained similar (Watch PR 1.11, 95% CI 1.04–1.19; Reserve PR 1.26, 95% CI 1.12–1.43; Table S8).

## 4 Discussion

Our findings indicate that antibiotic dosing‐related errors are a substantial component of medication errors reported to VigiMed and that they occur across different antibiotic classes and age groups. DEPs accounted for approximately one‐third of antibiotic MEPs (*n* = 2587), with dose omission and dosing regimen problems being the leading subtypes. A higher proportion of drug–event pairs was classified as MEPs at the extremes of age, and the proportion of DEPs was higher among Watch and Reserve antibiotics. Consistent with population‐ and hospital‐based studies showing that anti‐infectives frequently rank among therapeutic classes involved in preventable events, dosing‐related errors involving systemic antibiotics appear to represent a recurrent problem across healthcare settings [19, 20, 43]. Similar patterns have been reported in the Russian pharmacovigilance database, where nearly one‐third of medication errors involving beta‐lactams concerned inappropriate dose or regimen, comparable to the present findings [44].

Because spontaneous reporting systems are subject to underreporting [45], the number of DEPs identified in our study likely underestimates the true burden of these preventable events. Dosing‐related errors are relevant to both patient safety and antimicrobial stewardship because they may contribute to AMR and may be addressed through targeted stewardship interventions.

Regarding DEP classification (Table 1), errors consistent with subtherapeutic exposure—dose omission (51.4%) and subtherapeutic dose (5.5%)—accounted for 56.9% of DEPs. Within this distribution, the predominance of dose omission was amplified by its higher proportion among pairs coded as ‘medicine not administered’ (67.2%) than among those coded as ‘suspect’ (44.4%). Such errors may compromise treatment effectiveness and pharmacokinetic/pharmacodynamic target attainment and represent an important stewardship concern [5, 6, 46]. From a One Health perspective, both subtherapeutic exposure and overdosing may have consequences beyond prolonged hospitalisation or failure to achieve clinical cure, including broader ecological and public health implications for AMR [3, 6, 47]. Evidence on the frequency and clinical impact of delayed and subtherapeutic doses remains limited [48, 49], warranting further study to inform stewardship interventions.

The contextual analysis explored demographic and reporting characteristics associated with medication‐error classification within VigiMed (Figure 2). Compared with adults, the adjusted prevalence of medication‐error classification was higher among neonates (adjusted PR 3.90), infants (1.78) and older adults (1.70), consistent with previous reports of greater medication‐use complexity at the extremes of age [9, 10, 11, 46, 50, 51]. Pairs reported through non‐healthcare sources had a lower adjusted prevalence, possibly reflecting the Brazilian framework, under which reporting is required for both healthcare services and marketing authorisation holders [26, 27]. As this analysis combined dosing‐ and non‐dosing‐related errors, its findings provide context for patterns of medication‐error reporting within VigiMed. Missing demographic information may have affected the adjusted estimates; therefore, these associations should be interpreted cautiously.

Watch antibiotics accounted for most MEPs. Widely used hospital antibiotics such as ceftriaxone, vancomycin, meropenem and piperacillin/tazobactam were among the most frequently reported agents and were associated with DEPs across age groups. This finding probably reflects the widespread use of these antibiotics in healthcare settings. Several of the most frequently reported Watch agents were broad‐spectrum beta‐lactams, for which delays, dose omissions and subtherapeutic exposure may compromise pharmacokinetic/pharmacodynamic target attainment [4, 52, 53, 54, 55, 56, 57]. A study of AMR trends and inappropriate antibiotic use in lower‐middle‐income countries, based on data from VigiBase, similarly found a predominance of reported suspected events involving Watch agents (*n =* 1739; 58.8%) [16]. Although reporting propensity may differ across antibiotics, particularly because some agents are subject to closer monitoring in hospital settings, these findings highlight challenges in the appropriate use of antibiotics in routine care in Brazil.

The heatmap (Figure 3) shows the proportion of DEPs across antibiotic–age‐group strata relative to reporting volume. This exploratory distribution may support hypothesis generation and prioritisation of dose‐optimisation strategies. It should be noted that some strata contained few drug–event pairs, and extreme proportions may reflect sparse data. Such surveillance‐oriented displays may help identify antibiotic–age‐group combinations warranting closer review of dosing and prescribing practices, particularly in local surveillance over time.

The AWaRe classification allows DEPs to be examined across stewardship‐relevant groups, and the adjusted estimates particularly highlight Reserve antibiotics, despite the intensive monitoring typically applied to these last‐resort agents, with a 31% higher proportion of DEPs among MEPs than Access antibiotics (adjusted PR 1.31; 95% CI 1.16–1.49), followed by Watch antibiotics (1.14, 1.07–1.22). This analysis may therefore help identify routine dosing and prescribing practices requiring closer scrutiny. However, these associations may reflect clinical context, including infection severity, hospital and critical care use, multidrug‐resistant infections and therapeutic monitoring requirements, as well as differential reporting and require further investigation. In spontaneous reporting systems, these associations should not be interpreted as causal.

Estimates were slightly attenuated but remained stable when the analysis was restricted to ‘suspect’ drug–event pairs (Watch 1.10, 1.01–1.19; Reserve 1.23, 1.05–1.44) and after adjustment for calendar year (Watch 1.11, 1.04–1.19; Reserve 1.26, 1.12–1.43). This consistency across different drug–event relationships and temporal adjustment supports the robustness of the observed association.

Given the recognised limitations of spontaneous reporting systems, the adjusted models were used primarily to assess the consistency of patterns within the reported data. The main contribution of this study is descriptive, characterising MEPs and DEPs within VigiMed and highlighting the value of pharmacovigilance databases in revealing recurrent vulnerabilities in medication use that may not be captured in controlled settings [58].

Pharmacovigilance databases are increasingly used to generate AMR‐relevant insights [16, 18, 59, 60, 61, 62]. Spontaneous reporting systems capture inappropriate antibiotic use, including prescribing errors, dosing problems and deviations from clinical recommendations [17]. Our analysis may inform targeted antimicrobial stewardship and regulatory action and demonstrate how pharmacovigilance data can support safe use of medicines. Complementary study designs are needed to quantify the burden and clinical impact of dosing‐related errors in Brazil.

Wider use of interoperable, structured information systems linking prescribing, dispensing and adverse event reporting could enable systematic monitoring. Artificial intelligence–based dosing support also shows promise [63, 64, 65, 66, 67, 68], but its integration into Brazilian healthcare requires further evaluation. Strategies to improve antibiotic use include greater involvement of clinical pharmacists, electronic prescribing and structured antimicrobial stewardship programmes, all of which may reduce medication errors.

### 4.1 Limitations and Strengths

The analyses were based on reported events rather than antibiotic exposure. Prevalence ratios do not reflect incidence, absolute risk, or the true frequency of dosing‐related errors among antibiotic users. Reporting propensity may also differ across antibiotics, potentially influencing the relative frequency of reported DEPs for specific agents. Thus, results should be interpreted as reflecting reported events and reporting patterns rather than population‐level measures of antibiotic‐related risk.

The high proportion of ‘Not reported/Unknown’ values limited the analysis of certain variables, including event seriousness. Missing values in spontaneous reporting systems are unlikely to occur completely at random and may reflect differences in reporter type, reporting practices, event severity, healthcare setting or information availability at the time of reporting. Missing categories were retained for transparency, but their estimates should be interpreted cautiously, as they may reflect information bias rather than actual differences in the distribution of DEPs.

Reports containing multiple antibiotics or events contributed more than one drug–event pair and could therefore disproportionately influence counts, rankings and heatmap patterns. Most reports involved a single antibiotic and a single event (median 1.0 for both; means 1.05 and 1.18, respectively), which limited but did not eliminate this potential influence. Although regression models accounted for clustering at the report level, the descriptive analyses did not and their findings should be interpreted accordingly.

Despite these limitations, this study shows how a large national pharmacovigilance database can characterise reported medication‐use problems and identify recurrent vulnerabilities in antibiotic use. Although such analyses cannot establish causality or estimate population‐level risk, they can strengthen other surveillance approaches and support antimicrobial stewardship in Brazil and similar settings.

## 5 Conclusion

Data from VigiMed indicate that DEPs accounted for nearly one‐third of antibiotic‐associated MEPs reported to the national pharmacovigilance system in Brazil. Dose omissions and dosing regimen problems were the most frequently reported subtypes, followed by incorrect dose, overdose and subtherapeutic dose. Reported errors involving Watch and Reserve antibiotics—agents typically subject to enhanced oversight—highlight clinically relevant vulnerabilities in antibiotic medication safety.

Integrating pharmacovigilance data into antimicrobial stewardship could help prioritise age groups, target dose‐optimisation efforts and strengthen regulatory oversight. Improving report completeness and clinical detail may make pharmacovigilance data easier to interpret and more useful for decision‐making. At the service level, systematic review of aggregated local reports may identify recurrent process failures, support feedback to prescribers and care teams and guide targeted interventions to improve antibiotic use practices. Further studies linking reported dosing‐related errors with clinical outcomes and AMR patterns are needed to provide a stronger evidence base for targeted antimicrobial stewardship and medication‐safety interventions in Brazil.

## Author Contributions

**Valcieny Sandes**, **Alice Ramos‐Silva**, **Albert Figueras** and **Elisangela Costa Lima:** conceptualization. **Valcieny Sandes:** data curation. **Valcieny Sandes** and **Alice Ramos‐Silva:** formal analysis. **Valcieny Sandes**, **Adriana Ivama‐Brummell**, **Albert Figueras** and **Elisangela Costa Lima:** methodology. **Albert Figueras** and **Elisangela Costa Lima:** supervision. **Valcieny Sandes:** writing – original draft. **Alice Ramos‐Silva**, **Adriana Ivama‐Brummell**, **Albert Figueras** and **Elisangela Costa Lima:** writing – review and editing. All authors have read and agreed to the final version of the manuscript.

## Funding

This work was supported by a grant from the Fundação Carlos Chagas Filho de Amparo à Pesquisa do Estado do Rio de Janeiro (FAPERJ; E‐26/201.373/2022). The sponsor had no role in the study design, data collection, analysis, or writing of the manuscript.

## Disclosure

The views expressed in this article are those of the authors and do not necessarily reflect those of the Brazilian Health Regulatory Agency (Anvisa).

## Ethics Statement

This study relied exclusively on secondary data from a publicly available open‐access database. The dataset contains no personal identifiers or sensitive information. Therefore, submission to a Research Ethics Committee was not required.

## Conflicts of Interest

The authors declare no conflicts of interest.

## Supporting information


**Table S1:** Data completeness of study variables in the VigiMed database (Brazil, 2018–2025).
**Table S2:** Terminological harmonisation of antibacterial drugs reported in VigiMed (Brazil, 2018–2025).
**Table S3:** Characteristics of systemic antibacterial (ATC J01) drug–event pairs by error classification, VigiMed (Brazil, 2018–2025).
**Table S4:** Frequency of antibiotics associated with dosing‐related error drug–event pairs in VigiMed (Brazil, 2018–2025, *n* = 2587).
**Table S5:** Crude and adjusted prevalence ratios for medication‐error classification among J01 drug–event pairs, VigiMed (Brazil, 2018–2025).
**Table S6:** Crude and adjusted prevalence ratios for the association between AWaRe antibiotic classification and dosing‐related error classification among medication‐error drug–event pairs, VigiMed (Brazil, 2018–2025).
**Table S7:** Sensitivity analysis: association between AWaRe class and dosing‐related error classification among medication‐error drug–event pairs restricted to a ‘suspect’ drug–event relationship, VigiMed (Brazil, 2018–2025).
**Table S8:** Sensitivity analysis: adjusted prevalence ratios for the association between AWaRe classification and dosing‐related error classification among medication‐error drug–event pairs, additionally adjusted for calendar year of entry into the system, VigiMed (Brazil, 2018–2025).
**Table S9:** STROBE checklist for cross‐sectional studies.

**Supplementary Methods S1:** Detailed deduplication algorithm.
**Supplementary Methods S2:** Variable harmonisation.
**Supplementary Methods S3:** Detailed analytical selection criteria.
**Supplementary Methods Table 1:** Dosing‐related error terms identified in the study dataset and their classification (Brazil, VigiMed, 2018–2025).

## Data Availability

The VigiMed data extracts used in this study, downloaded in December 2025 from the Brazilian Health Regulatory Agency (Anvisa) Pharmacovigilance Reporting Database and accessible through the Open Data Portal [29], are publicly available on Zenodo at https://doi.org/10.5281/zenodo.21895098. The analysis code, harmonisation file, software requirements and supporting documentation are publicly available at https://doi.org/10.5281/zenodo.22815748. The deposited files comprise the data‐processing pipeline (data cleaning, deduplication, linkage, harmonisation and selection of eligible antibiotic drug–event pairs) and the statistical analyses. The MedDRA SMQ for Medication Error file is required at the pipeline step that generates the medication‐error and dosing‐related error datasets. The statistical analyses can be reproduced without this file, using the intermediate antibiotic dataset and the PT lists included in the code (32 dosing‐related and 189 other medication‐error PTs).

## References

[1] B. G. Bell , F. Schellevis , E. Stobberingh , H. Goossens , and M. Pringle , “A Systematic Review and Meta‐Analysis of the Effects of Antibiotic Consumption on Antibiotic Resistance,” BMC Infectious Diseases 14 (2014): 13, 10.1186/1471-2334-14-13. PMC3897982 24405683

[2] S. W. Olesen , M. Lipsitch , and Y. H. Grad , “The Role of “Spillover” in Antibiotic Resistance,” Proceedings of the National Academy of Sciences of the United States of America 117, no. 46 (2020): 29063–29068, 10.1073/pnas.2013694117. PMC7682407 33139558

[3] J. A. Roberts , P. Kruger , D. L. Paterson , and J. Lipman , “Antibiotic Resistance—What's Dosing Got to Do With It?,” Critical Care Medicine 36, no. 8 (2008): 2433–2440, 10.1097/CCM.0b013e318180fe62. 18596628

[4] F. Serapide , S. Rotundo , L. Gallelli , et al., “The Changing Landscape of Antibiotic Treatment: Reevaluating Treatment Length in the Age of New Agents,” Antibiotics 14, no. 7 (2025): 727, 10.3390/antibiotics14070727. PMC12291801 40724028

[5] L. G. Osterberg , J. Urquhart , and T. F. Blaschke , “Understanding Forgiveness: Minding and Mining the Gaps Between Pharmacokinetics and Therapeutics,” Clinical Pharmacology and Therapeutics 88, no. 4 (2010): 457–459, 10.1038/clpt.2010.171. 20856243

[6] C. N. Patel , M. D. Swartz , J. S. Tomasek , L. E. Vincent , W. E. Hallum , and J. B. Holcomb , “The Effects of Missed Doses of Antibiotics on Hospitalized Patient Outcomes,” Journal of Surgical Research 233 (2019): 276–283, 10.1016/j.jss.2018.08.015. 30502260

[7] D. H. Cousins , D. Gerrett , and B. Warner , “A Review of Medication Incidents Reported to the National Reporting and Learning System in England and Wales Over 6 Years (2005–2010),” British Journal of Clinical Pharmacology 74, no. 4 (2012): 597–604, 10.1111/j.1365-2125.2011.04166.x. PMC3477327 22188210

[8] J. A. Villalobos‐Madriz , E. Zavaleta‐Monestel , E. Martínez‐Vargas , et al., “Medication Error Management in Private Hospitals in Costa Rica: A Mixed‐Methods Evaluation of Practices and Improvement Strategies,” Cureus 17, no. 2 (2025): e78693, 10.7759/cureus.78693. PMC11890543 40062057

[9] A. A. Alghamdi , R. N. Keers , A. Sutherland , and D. M. Ashcroft , “Prevalence and Nature of Medication Errors and Preventable Adverse Drug Events in Paediatric and Neonatal Intensive Care Settings: A Systematic Review,” Drug Safety 42, no. 12 (2019): 1423–1436, 10.1007/s40264-019-00856-9. PMC6858386 31410745

[10] G. D. Hannibal , N. Vithanage , M. T. Madhushika , T. K. Sinhabahu , I. Kankananarachchi , and P. Liyanage , “A Systematic Review of Prescription Errors in Paediatric Care,” BMC Health Services Research 25, no. 1 (2025): 967, 10.1186/s12913-025-13109-6. PMC12281725 40696335

[11] A. Kaligirwa , E. L. Peter , and P. E. Alele , “Medication Errors Among Elderly In‐Patients at an Urban National Referral Hospital in Tanzania: Prevalence and Patient Profiles in a Low‐Resource Setting,” African Health Sciences 26, no. 1 (2026): 151–163, 10.4314/ahs.v26i1.16. PMC13126145 42063905

[12] N. Carral , J. C. Lukas , I. Oteo , and E. Suarez , “Impact of Poor Compliance With Levofloxacin and Moxifloxacin on Respiratory Tract Infection Antimicrobial Efficacy: A Pharmacokinetic/Pharmacodynamic Simulation Study,” International Journal of Antimicrobial Agents 45, no. 1 (2015): 79–83, 10.1016/j.ijantimicag.2014.08.011. 25450804

[13] A. H. Holmes , L. S. P. Moore , A. Sundsfjord , et al., “Understanding the Mechanisms and Drivers of Antimicrobial Resistance,” Lancet 387, no. 10014 (2016): 176–187, 10.1016/S0140-6736(15)00473-0. 26603922

[14] World Health Organization , “Pharmacovigilance,” accessed June 18, 2026, https://www.who.int/teams/regulation‐prequalification/regulation‐and‐safety/pharmacovigilance.

[15] European Medicines Agency , “Pharmacovigilance: Overview,” accessed June 18, 2026, https://www.ema.europa.eu/en/human‐regulatory‐overview/pharmacovigilance‐overview.

[16] H. Saleh , J. Mitchell , C. S. Lundborg , and M. Sharma , “The Role of Pharmacovigilance Database in Identifying Antibiotic Resistance and Inappropriate Use: An Analysis of VigiBase Reports From Lower‐Middle‐Income Countries,” Pharmacoepidemiology and Drug Safety 35, no. 6 (2026): e70404, 10.1002/pds.70404. PMC13247192 42260726

[17] V. Sandes , A. Figueras , and E. C. Lima , “Pharmacovigilance Strategies to Address Resistance to Antibiotics and Inappropriate Use—A Narrative Review,” Antibiotics 13, no. 5 (2024): 457, 10.3390/antibiotics13050457. PMC11117530 38786184

[18] J. M. V. Habarugira and A. Figueras , “Pharmacovigilance Network as an Additional Tool for the Surveillance of Antimicrobial Resistance,” Pharmacoepidemiology and Drug Safety 30, no. 8 (2021): 1123–1131, 10.1002/pds.5249. 33864401

[19] World Health Organization , Global Burden of Preventable Medication‐Related Harm in Health Care: A Systematic Review (World Health Organization, 2023), accessed February 20, 2026, https://iris.who.int/server/api/core/bitstreams/f208903d‐b47d‐4a8c‐9fac‐d8136c2bdbb6/content.

[20] L. Assunção‐Costa , I. Costa de Sousa , M. R. Alves de Oliveira , et al., “Drug Administration Errors in Latin America: A Systematic Review,” PLoS One 17, no. 8 (2022): e0272123, 10.1371/journal.pone.0272123. PMC9352042 35925985

[21] M. Salas , L. C. Lopes , B. Godman , et al., “Challenges Facing Drug Utilization Research in the Latin American Region,” Pharmacoepidemiology and Drug Safety 29, no. 11 (2020): 1353–1363, 10.1002/pds.4989. 32419226

[22] L. F. Leal , C. G. S. Osorio‐de‐Castro , L. J. C. de Souza , et al., “Data Sources for Drug Utilization Research in Brazil‐DUR‐BRA Study,” Frontiers in Pharmacology 12 (2022): 789872, 10.3389/fphar.2021.789872. PMC8805708 35115935

[23] N. Restrepo‐Arbeláez , J. C. Garcia‐Betancur , C. J. Pallares , and M. V. Villegas , “Antimicrobial Stewardship Programs in Latin America and the Caribbean: A Story of Perseverance, Challenges, and Goals,” Antibiotics 12, no. 8 (2023): 1342, 10.3390/antibiotics12081342. PMC10452019 37627762

[24] World Health Organization , “AWaRe Classification of Antibiotics for Evaluation and Monitoring of Use,” 2023, accessed August 29, 2025, https://www.who.int/publications/i/item/WHO‐MHP‐HPS‐EML‐2023.04.

[25] Agência Nacional de Vigilância Sanitária (Anvisa) , “VigiMed,” accessed July 14, 2025, https://www.gov.br/anvisa/pt‐br/assuntos/fiscalizacao‐e‐monitoramento/notificacoes/vigimed/capa‐vigimed.

[26] Brasil. Ministério da Saúde. Agência Nacional de Vigilância Sanitária , “Resolução da Diretoria Colegiada ‐ RDC n^o^ 36, de 25 de julho de 2013,” accessed February 19, 2026, https://anvisalegis.datalegis.net/action/ActionDatalegis.php?acao=abrirTextoAto&tipo=RDC&numeroAto=00000036&seqAto=000&valorAno=2013&orgao=RDC/DC/ANVISA/MS&codTipo=&desItem=&desItemFim=&cod_menu=1696&cod_modulo=134&pesquisa=true.

[27] Brasil. Ministério da Saúde. Agência Nacional de Vigilância Sanitária , “Resolução da Diretoria Colegiada ‐ RDC n^o^ 406, de 22 de julho de 2020,” accessed February 19, 2026, https://anvisalegis.datalegis.net/action/ActionDatalegis.php?acao=abrirTextoAto&tipo=RDC&numeroAto=00000406&seqAto=000&valorAno=2020&orgao=RDC/DC/ANVISA/MS&codTipo=&desItem=&desItemFim=&cod_menu=1696&cod_modulo=134&pesquisa=true.

[28] WHO Collaborating Centre for Drug Statistics Methodology , “ATC/DDD Index,” accessed June 18, 2025, https://atcddd.fhi.no/atc_ddd_index/.

[29] Agência Nacional de Vigilância Sanitária (Anvisa) , “Notificações em Farmacovigilância,” Portal Brasileiro de Dados Abertos, accessed January 18, 2026, https://dados.gov.br/dados/conjuntos‐dados/notificacoes‐em‐farmacovigilancia.

[30] Uppsala Monitoring Centre , “WHODrug Global,” accessed June 18, 2025, https://who‐umc.org/whodrug/whodrug‐global/.

[31] MedDRA , “MedDRA Maintenance and Support Services Organization. Medical Dictionary for Regulatory Activities (MedDRA),” accessed June 18, 2025, https://www.meddra.org/.

[32] G. N. Norén , R. Orre , A. Bate , and I. R. Edwards , “Duplicate Detection in Adverse Drug Reaction Surveillance,” Data Mining and Knowledge Discovery 14, no. 3 (2007): 305–328, 10.1007/s10618-006-0052-8.

[33] R. Kiguba , G. Isabirye , J. Mayengo , et al., “Navigating Duplication in Pharmacovigilance Databases: A Scoping Review,” BMJ Open 14, no. 4 (2024): e081990, 10.1136/bmjopen-2023-081990. PMC11086478 38684275

[34] P. M. Tregunno , D. B. Fink , C. Fernandez‐Fernandez , E. Lázaro‐Bengoa , and G. N. Norén , “Performance of Probabilistic Method to Detect Duplicate Individual Case Safety Reports,” Drug Safety 37, no. 4 (2014): 249–258, 10.1007/s40264-014-0146-y. 24627310

[35] Agência Nacional de Vigilância Sanitária (Anvisa) , “VigiMed: Perguntas e Respostas,” accessed June 24, 2025, https://www.gov.br/anvisa/pt‐br/assuntos/fiscalizacao‐e‐monitoramento/notificacoes/vigimed/arquivos/vigimed‐perguntas‐e‐respostas.pdf/view.

[36] MedDRA , “Standardised MedDRA Queries (SMQs),” accessed January 22, 2026, https://www.meddra.org/how‐to‐use/tools/smqs.

[37] National Coordinating Council for Medication Error Reporting and Prevention , “Medication Error Definition,” accessed June 18, 2026, https://www.nccmerp.org/about‐medication‐errors.

[38] Uppsala Monitoring Centre , “Entrada de Dados em VigiFlow,” accessed June 25, 2025, https://who‐umc.org/vigiverse/pv‐product‐support/entrada‐de‐dados/.

[39] G. Zou , “A Modified Poisson Regression Approach to Prospective Studies With Binary Data,” American Journal of Epidemiology 159, no. 7 (2004): 702–706, 10.1093/aje/kwh090. 15033648

[40] L. N. Yelland , A. B. Salter , and P. Ryan , “Performance of the Modified Poisson Regression Approach for Estimating Relative Risks From Clustered Prospective Data,” American Journal of Epidemiology 174, no. 8 (2011): 984–992, 10.1093/aje/kwr183. 21841157

[41] G. Y. Zou and A. Donner , “Extension of the Modified Poisson Regression Model to Prospective Studies With Correlated Binary Data,” Statistical Methods in Medical Research 22, no. 6 (2013): 661–670, 10.1177/0962280211427759. 22072596

[42] E. von Elm , D. G. Altman , M. Egger , S. J. Pocock , P. C. Gøtzsche , and J. P. Vandenbroucke , “The Strengthening the Reporting of Observational Studies in Epidemiology (STROBE) Statement: Guidelines for Reporting Observational Studies,” Journal of Clinical Epidemiology 61, no. 4 (2008): 344–349, 10.1016/j.jclinepi.2007.11.008. 18313558

[43] M. Tobaiqy and K. MacLure , “Medication Errors in Saudi Arabian Hospital Settings: A Systematic Review,” Medicina 60, no. 9 (2024): 1411, 10.3390/medicina60091411. PMC11433733 39336452

[44] A. V. Kuzmina , I. L. Asetskaya , S. K. Zyryanov , and V. A. Polivanov , “Detecting Medication Errors Associated With the Use of Beta‐Lactams in the Russian Pharmacovigilance Database,” BMC Pharmacology and Toxicology 22, no. 1 (2021): 5, 10.1186/s40360-020-00470-x. PMC7796638 33419474

[45] P. García‐Abeijon , C. Costa , M. Taracido , M. T. Herdeiro , C. Torre , and A. Figueiras , “Factors Associated With Underreporting of Adverse Drug Reactions by Health Care Professionals: A Systematic Review Update,” Drug Safety 46, no. 7 (2023): 625–636, 10.1007/s40264-023-01302-7. PMC10279571 37277678

[46] G. Fu , W. Sun , Z. Tan , B. Liang , and Y. Cai , “An Insight Into Pharmacokinetics and Dose Optimization of Antimicrobials Agents in Elderly Patients,” Frontiers in Pharmacology 15 (2024): 1396994, 10.3389/fphar.2024.1396994. PMC11471682 39403139

[47] B. Aslam , M. Khurshid , M. I. Arshad , et al., “Antibiotic Resistance: One Health One World Outlook,” Frontiers in Cellular and Infection Microbiology 11 (2021): 771510, 10.3389/fcimb.2021.771510. PMC8656695 34900756

[48] T. Pullam , C. L. Russell , and S. White‐Lewis , “Frequency of Medication Administration Timing Error in Hospitals: A Systematic Review,” Journal of Nursing Care Quality 38, no. 2 (2023): 126–133, 10.1097/NCQ.0000000000000668. 36332227

[49] S. Rajakumar , R. Rajah , S. Thanimalai , F. B. M. Mokhtar , and D. S. Ramachandram , “Intravenous Medication Administration Errors in Hospitalised Patients: An Updated Systematic Review,” Journal of Evaluation in Clinical Practice 31, no. 4 (2025): e70167, 10.1111/jep.70167. PMC12183495 40545841

[50] L. Soraci , A. Cherubini , L. Paoletti , et al., “Safety and Tolerability of Antimicrobial Agents in the Older Patient,” Drugs & Aging 40, no. 6 (2023): 499–526, 10.1007/s40266-023-01019-3. PMC10043546 36976501

[51] N. D. Rivera‐Chaparro , M. Cohen‐Wolkowiez , and R. G. Greenberg , “Dosing Antibiotics in Neonates: Review of the Pharmacokinetic Data,” Future Microbiology 12, no. 11 (2017): 1001–1016, 10.2217/fmb-2017-0058. PMC5627030 28758800

[52] M. S. Alikhani , M. Nazari , and S. Hatamkhani , “Enhancing Antibiotic Therapy Through Comprehensive Pharmacokinetic/Pharmacodynamic Principles,” Frontiers in Cellular and Infection Microbiology 15 (2025): 1521091, 10.3389/fcimb.2025.1521091. PMC11893874 40070375

[53] E. Asín‐Prieto , A. Rodríguez‐Gascón , and A. Isla , “Applications of the Pharmacokinetic/Pharmacodynamic (PK/PD) Analysis of Antimicrobial Agents,” Journal of Infection and Chemotherapy 21, no. 5 (2015): 319–329, 10.1016/j.jiac.2015.02.001. 25737147

[54] T. R. Perry and J. J. Schentag , “Clinical Use of Ceftriaxone: A Pharmacokinetic‐Pharmacodynamic Perspective on the Impact of Minimum Inhibitory Concentration and Serum Protein Binding,” Clinical Pharmacokinetics 40, no. 9 (2001): 685–694, 10.2165/00003088-200140090-00004. 11605716

[55] N. J. Onufrak , A. Forrest , and D. Gonzalez , “Pharmacokinetic and Pharmacodynamic Principles of Anti‐Infective Dosing,” Clinical Therapeutics 38, no. 9 (2016): 1930–1947, 10.1016/j.clinthera.2016.06.015. PMC5039113 27449411

[56] J. M. Varghese , J. A. Roberts , and J. Lipman , “Antimicrobial Pharmacokinetic and Pharmacodynamic Issues in the Critically Ill With Severe Sepsis and Septic Shock,” Critical Care Clinics 27, no. 1 (2011): 19–34, 10.1016/j.ccc.2010.09.006. 21144984

[57] DrugBank Online , “Browsing Drugs,” accessed September 4, 2025, https://go.drugbank.com/drugs.

[58] World Health Organization , The Importance of Pharmacovigilance: Safety Monitoring of Medicinal Products (World Health Organization, 2002), accessed April 11, 2026, https://www.who.int/publications/i/item/10665‐42493.

[59] J. Mitchell , C. Westerberg , M. Purohit , P. Lundquist , and C. Stålsby Lundborg , “The Enhancing Role of Pharmacovigilance to Conventional Antibiotic Resistance Surveillance: Cross‐Sectional Identification and Analysis of Reports of Antibiotic Resistance in VigiBase,” International Journal of Infectious Diseases 158 (2025): 107947, 10.1016/j.ijid.2025.107947. 40480533

[60] H. Lin , C. Zhu , S. Liu , Y. Bi , J. Hu , and M. Ju , “Post‐Market Safety Profile of Cefiderocol: A Real‐World Pharmacovigilance Exploratory Analysis Based on U.S. FDA Adverse Event Reporting System (FAERS),” BMC Pharmacology and Toxicology 26, no. 1 (2025): 58, 10.1186/s40360-025-00894-3. PMC11899456 40069825

[61] C. Cagnotta , A. Zinzi , F. Gargano , et al., “Can Pharmacovigilance Data Represent a Potential Tool for Early Detection of the Antibiotic Resistance Phenomenon?,” Pharmacoepidemiology 3, no. 4 (2024): 350–364, 10.3390/pharma3040024.

[62] A. M. Arseniu , B. I. Vintila , A. Butuca , et al., “Resistance, Ineffectiveness, and Off‐Label Use Related to Cephalosporins From the Reserve Group—A Pharmacovigilance Signal Detection Study on EudraVigilance Database,” Pharmaceuticals 19, no. 1 (2026): 155, 10.3390/ph19010155. PMC12844660 41599752

[63] M. D. Canales‐Siguero , C. García‐Muñoz , J. M. Caro‐Teller , et al., “Impact of Pharmaceutical Validation on Prescribing Errors in a Neonatal Intensive Care Unit. Randomised and Controlled Study,” Frontiers in Pediatrics 12 (2024): 1346090, 10.3389/fped.2024.1346090. PMC11025468 38638590

[64] J. D. Oliveira , H. D. P. Santos , A. H. D. P. S. Ulbrich , et al., “Development and Evaluation of a Clinical Note Summarization System Using Large Language Models,” Communications Medicine 5, no. 1 (2025): 376, 10.1038/s43856-025-01091-3. PMC12394402 40877595

[65] M. I. Tejeda , J. Fernández , P. Valledor , C. Almirall , J. Barberán , and S. Romero‐Brufau , “Retrospective Validation Study of a Machine Learning‐Based Software for Empirical and Organism‐Targeted Antibiotic Therapy Selection,” Antimicrobial Agents and Chemotherapy 68, no. 10 (2024): e0077724, 10.1128/aac.00777-24. PMC11460031 39194206

[66] H. Bilal , M. N. Khan , S. Khan , et al., “The Role of Artificial Intelligence and Machine Learning in Predicting and Combating Antimicrobial Resistance,” Computational and Structural Biotechnology Journal 27 (2025): 423–439, 10.1016/j.csbj.2025.01.006. PMC11791014 39906157

[67] S. Y. Park , “Leveraging Hospital Information Data for Effective Antibiotic Stewardship,” Journal of Korean Medical Science 40, no. 20 (2025): e163, 10.3346/jkms.2025.40.e163. PMC12105991 40425195

[68] C. Codde , J. F. Faucher , and J. B. Woillard , “Use of Artificial Intelligence in Current Fight Against Antimicrobial Resistance,” Microbial Drug Resistance 32, no. 10 (2025): 393–402, 10.1089/mdr.2024.0241. 40354275

