# Supplementary material for: Reported Dosing‐Related Medication Errors Involving Systemic Antibiotics in the Brazilian Pharmacovigilance Database (2018–2025): Distribution Across the WHO AWaRe Classification
Source: Pharmacoepidemiol Drug Saf. 2026 Oct 7;35(10):e70496. doi: 10.1002/pds.70496 (PMC13643816; doi:10.1002/pds.70496)
Supplement: Supplementary file 1 [file PDS-35-e70496-s001.docx]

Supporting Information

[Supplementary Table S1. Data completeness of study variables in the VigiMed database (Brazil, 2018–2025).	2](#_Toc238203310)

[Supplementary Table S2. Terminological harmonisation of antibacterial drugs reported in VigiMed (Brazil, 2018–2025).	3](#_Toc238203311)

[Supplementary Table S3. Characteristics of systemic antibacterial (ATC J01) drug–event pairs by error classification, VigiMed (Brazil, 2018–2025).	7](#_Toc238203312)

[Supplementary Figure S1. Distribution of the five most frequently reported antibiotics, associated with dosing-related error drug–event pairs within each age group, expressed as unadjusted percentages, VigiMed (Brazil, 2018-2025; *n =* 2,587)	8](#_Toc238203313)

[Supplementary Table S4. Frequency of antibiotics associated with dosing-related error drug–event pairs in VigiMed (Brazil, 2018–2025, *n* = 2,587)	9](#_Toc238203314)

[Supplementary Table S5. Crude and adjusted prevalence ratios for medication-error classification among J01 drug–event pairs, VigiMed (Brazil, 2018–2025).	11](#_Toc238203315)

[Supplementary Table S6. Crude and adjusted prevalence ratios for the association between AWaRe antibiotic classification and dosing-related error classification among medication-error drug–event pairs, VigiMed (Brazil, 2018–2025).	12](#_Toc238203316)

[Supplementary Table S7. Sensitivity analysis: association between AWaRe class and dosing-related error classification among medication-error drug–event pairs restricted to a “suspect” drug–event relationship, VigiMed (Brazil, 2018–2025).	13](#_Toc238203317)

[Supplementary Table S8. Supplementary Table S8. Sensitivity analysis: adjusted prevalence ratios for the association between AWaRe classification and dosing-related error classification among medication-error drug–event pairs, additionally adjusted for calendar year of entry into the system, VigiMed (Brazil, 2018–2025).	14](#_Toc238203318)

[Supplementary Figure S2. Dosing-related error drug–event pairs by year and AWaRe class, 2018–2025	15](#_Toc238203319)

[Supplementary Table S9. STROBE checklist for cross-sectional studies.	16](#_Toc238203320)

# Supplementary Table S1. Data completeness of study variables in the VigiMed database (Brazil, 2018–2025).

| **Variable** | **Non-missing, n** | **Non-missing, %** |
| --- | --- | --- |
| UF | 225544 | 73.47 |
| TIPO_ENTRADA_VIGIMED | 306931 | 99.98 |
| RECEBIDO_DE | 252273 | 82.17 |
| IDENTIFICACAO_NOTIFICACAO | 307001 | 100.0 |
| DATA_INCLUSAO_SISTEMA | 307001 | 100.0 |
| DATA_ULTIMA_ATUALIZACAO | 306935 | 99.98 |
| DATA_NOTIFICACAO | 157150 | 51.19 |
| TIPO_NOTIFICACAO | 307001 | 100.0 |
| NOTIFICACAO_PARENT_CHILD | 1114 | 0.36 |
| DATA_NASCIMENTO | 227394 | 74.07 |
| IDADE_MOMENTO_REACAO | 197675 | 64.39 |
| GRUPO_IDADE | 125303 | 40.82 |
| IDADE_GESTACIONAL_MOMENTO_REACAO | 328 | 0.11 |
| SEXO | 295478 | 96.25 |
| GESTANTE | 146189 | 47.62 |
| LACTANTE | 146186 | 47.62 |
| PESO_KG | 120219 | 39.16 |
| ALTURA_CM | 65773 | 21.42 |
| REACAO_EVENTO_ADVERSO_MEDDRA | 306685 | 99.9 |
| GRAVE | 238546 | 77.7 |
| GRAVIDADE | 140652 | 45.81 |
| DESFECHO | 257682 | 83.94 |
| DATA_INICIO_HORA | 212468 | 69.21 |
| DATA_FINAL_HORA | 130574 | 42.53 |
| DURACAO | 74132 | 24.15 |
| RELACAO_MEDICAMENTO_EVENTO | 307000 | 100.0 |
| NOME_MEDICAMENTO_WHODRUG | 306486 | 99.83 |
| ACAO_ADOTADA | 182626 | 59.49 |
| NOTIFICADOR | 289299 | 94.23 |

# Supplementary Table S2. Terminological harmonisation of antibacterial drugs reported in VigiMed (Brazil, 2018–2025).

| **Harmonised name** | **Active ingredients (WHODrug)** |
| --- | --- |
| Acriflavinium chloride \|Atropa belladonna \|Methenamine \|Methylthioninium chloride | Acriflavinium chloride \|Atropa belladonna \|Methenamine \|Methylthioninium chloride |
| Ambroxol\|Amoxicillin | Cloridrato de ambroxol_x000D_\|Amoxicilina trihidrato |
| Amikacin | Amicacina, Amikacin, Amikacin sulfate, Sulfato de amicacina |
| Amoxicillin | Amoxicilina sódica, Amoxicilina trihidrato, Amoxicillin, Amoxicillin sodium, Amoxicillin trihydrate |
| Amoxicillin\|Clarithromycin\|Esomeprazole | Amoxicilina trihidrato_x000D_\|Clarithromycin_x000D_\|Esomeprazol magnésico, Amoxicillin trihydrate\|Clarithromycin\|Esomeprazole magnesium, Amoxicillin\|Clarithromycin\|Esomeprazole |
| Amoxicillin\|Clarithromycin\|Lansoprazole | Amoxicilina trihidrato_x000D_\|Clarithromycin_x000D_\|Lansoprazole, Amoxicillin trihydrate\|Clarithromycin\|Lansoprazole |
| Amoxicillin\|Clarithromycin\|Omeprazole | Amoxicilina trihidrato_x000D_\|Clarithromycin_x000D_\|Omeprazole, Amoxicillin trihydrate\|Clarithromycin\|Omeprazole |
| Amoxicillin\|Clavulanic acid | Amoxicilina sódica_x000D_ \|Clavulanato de potássio, Amoxicilina trihidrato_x000D_ \|Ácido clavulânico, Amoxicilina trihidrato_x000D_ \|Clavulanato de potássio, Amoxicillin sodium \|Clavulanate potassium, Amoxicillin trihydrate \|Clavulanate potassium, Amoxicillin trihydrate \|Clavulanic acid, Amoxicillin_x000D_ \|Ácido clavulânico, Amoxicillin_x000D_ \|Clavulanato de potássio, Amoxicillin \|Clavulanate potassium, Amoxicillin \|Clavulanic acid |
| Amoxicillin\|Flucloxacillin | Amoxicillin trihydrate\|Flucloxacillin sodium |
| Amoxicillin\|Lansoprazole\|Levofloxacin | Amoxicillin trihydrate\|Lansoprazole\|Levofloxacin |
| Amoxicillin\|Sulbactam | Amoxicilina sódica_x000D_\|Sulbactam sódico, Amoxicilina trihidrato_x000D_\|Sulbactam pivoxila, Amoxicillin sodium \|Sulbactam sodium, Amoxicillin_x000D_ \|Sulbactam, Amoxicillin \|Sulbactam |
| Amphotericin b\|Tetracycline | Amphotericin b\|Tetracycline hydrochloride |
| Ampicillin | Ampicilina, Ampicilina sódica, Ampicilina trihidratada, Ampicillin, Ampicillin potassium, Ampicillin sodium, Ampicillin trihydrate |
| Ampicillin\|Sulbactam | Ampicilina sódica_x000D_ \|Sulbactam sódico, Ampicilina_x000D_ \|Sulbactam, Ampicillin sodium \|Sulbactam sodium, Ampicillin \|Sulbactam, Ampicilina\|Sulbactam |
| Avibactam | Avibactam |
| Avibactam\|Ceftazidime | Avibactam sódico_x000D_\|Ceftazidime, Avibactam sodium \|Ceftazidime, Avibactam sodium\|Ceftazidime pentahydrate, Avibactam_x000D_\|Ceftazidime, Avibactam\|Ceftazidime, Avibactam |
| Azithromycin | Azithromycin, Azithromycin dihydrate, Azithromycin monohydrate, Azitromicina di-hidratada, Azitromicina monoidratada |
| Aztreonam | Aztreonam |
| Bacitracin | Bacitracin |
| Benzathine benzylpenicillin | Benzathine benzylpenicillin, Benzilpenicilina benzatina, penicilina g benzatina |
| Benzathine benzylpenicillin\|Benzylpenicillin \|Procaine benzylpenicillin | Benzathine benzylpenicillin\|Benzylpenicillin potassium \|Procaine benzylpenicillin, Benzathine benzylpenicillin \|Benzylpenicillin sodium \|Procaine benzylpenicillin, Benzilpenicilina benzatina, penicilina g benzatina_x000D_ \|Benzilpenicilina potássica_x000D_\|Benzilpenicilina procaína, Benzilpenicilina benzatina, penicilina g benzatina_x000D_ \|Benzylpenicillin sodium_x000D_\|Benzilpenicilina procaína |
| Benzylpenicillin | Benzilpenicilina, Benzilpenicilina potássica, Benzylpenicillin, Benzylpenicillin potassium, Benzylpenicillin sodium |
| Benzylpenicillin\|Procaine benzylpenicillin | Benzilpenicilina potássica_x000D_\|Benzilpenicilina procaína, Benzylpenicillin sodium_x000D_\|Benzilpenicilina procaína, Benzylpenicillin sodium\|Procaine benzylpenicillin |
| Cefaclor | Cefaclor, Cefaclor monohydrate, Cefaclor monoidratado |
| Cefadroxil | Cefadroxil, Cefadroxil monohydrate, Cefadroxila, Cefadroxila monoidratada |
| Cefalexin | Cefalexin, Cefalexin hydrochloride, Cefalexin monohydrate, Cefalexin sodium, Cefalexina monoidratada |
| Cefalotin | Cefalotin, Cefalotin sodium, Cefalotina sódica |
| Cefazolin | Cefazolin, Cefazolin sodium, Cefazolina sódica |
| Cefcapene | Cefcapene pivoxil hydrochloride |
| Cefdinir | Cefdinir |
| Cefepime | Arginine\|Cefepime hydrochloride, Cefepime, Cefepime dihydrochloride monohydrate, Cefepime hydrochloride, Cloridrato de cefepima, L-arginina_x000D_\|Cloridrato de cefepima |
| Cefotaxime | Cefotaxima sódica, Cefotaxime, Cefotaxime sodium, Cefotaxime sodium\|Lidocaine hydrochloride |
| Cefoxitin | Cefoxitin, Cefoxitin sodium |
| Ceftaroline | Ceftaroline, Ceftaroline fosamil, Ceftaroline fosamil acetate |
| Ceftazidime | Ceftazidima pentaidratada, Ceftazidime, Ceftazidime pentahydrate |
| Ceftolozane\|Tazobactam | Ceftolozana_x000D_\|Tazobactam, Ceftolozane sulfate \|Tazobactam sodium, Ceftolozane\|Tazobactam, Sulfato de ceftolozana_x000D_\|Tazobactam sódico |
| Ceftriaxone | Ceftriaxona dissódica hemieptaidratada, Ceftriaxona sódica, Ceftriaxone, Ceftriaxone sodium, Ceftriaxone sodium sesquaterhydrate |
| Ceftriaxone\|Vancomycin | Ceftriaxone\|Vancomycin |
| Cefuroxime | Axetil cefuroxima, Cefuroxima sódica, Cefuroxime, Cefuroxime axetil, Cefuroxime sodium |
| Chloramphenicol | Chloramphenicol, Chloramphenicol palmitate, Chloramphenicol sodium succinate |
| Cilastatin\|Imipenem | Cilastatin sodium\|Imipenem, Cilastatin sodium\|Imipenem monohydrate, Cilastatin sodium\|Imipenem monohydrate \|Sodium bicarbonate, Cilastatin_x000D_\|Imipeném, Cilastatin\|Imipenem, Cilastatina sódica_x000D_\|Imipeném, Cilastatina sódica_x000D_\|Imipeném monoidratado |
| Ciprofloxacin | Ciprofloxacin, Ciprofloxacin hydrochloride, Ciprofloxacin hydrochloride monohydrate, Ciprofloxacin lactate, Cloridrato de ciprofloxacino, Cloridrato de ciprofloxacino monoidratado, Lactato de ciprofloxacino |
| Clarithromycin | Clarithromycin, Clarithromycin lactobionate |
| Clavulanic acid | Clavulanic acid |
| Clindamycin | Clindamycin, Clindamycin hydrochloride, Clindamycin palmitate hydrochloride, Clindamycin phosphate, Cloridrato de clindamicina, Cloridrato de palmitato de clindamicina, Fosfato de clindamicina |
| Cloxacillin | Cloxacillin |
| Colistin | Colistimethate sodium, Colistin, Colistin sulfomethate, Mesilato sódico de colistina |
| Daptomycin | Daptomycin |
| Delafloxacin | Delafloxacin, Delafloxacino meglumina |
| Doxycycline | Ácido ascórbico_x000D_\|Cloridrato de doxicilcina, Cloridrato de doxicilcina, Doxiciclina monoidratada, Doxycycline, Doxycycline calcium, Doxycycline hyclate, Doxycycline hydrochloride |
| Ertapenem | Ertapenem, Ertapeném sódico, Ertapenem sodium |
| Erythromycin | Erythromycin, Erythromycin estolate, Erythromycin ethylsuccinate, Erythromycin propionate, Estolato de eritromicina |
| Erythromycin\|Sulfafurazole | Erythromycin ethylsuccinate\|Sulfafurazole, Etilsuccinato de eritromicina_x000D_\|Sulfafurazole |
| Fosfomycin | Fosfomicina trometamol, Fosfomycin, Fosfomycin sodium, Fosfomycin trometamol |
| Gentamicin | Gentamicin, Gentamicin sulfate, Sulfato de gentamicina |
| Imipenem | Imipenem, Imipeném |
| Levofloxacin | Glucose\|Levofloxacin, Levofloxacin, Levofloxacin hemihydrate, Levofloxacina, Levofloxacino hemi-hidratado, levofloxacino hemiidratado |
| Linezolid | Linezolid, Linezolida |
| Loracarbef | Loracarbef |
| Lymecycline | Lymecycline |
| Meropenem | Meropenem, Meropeném, Meropenem sodium carbonate, Meropeném tri-hidratado, Meropenem trihydrate |
| Methenamine\|metiltionínio | Methenamine_x000D_\|Cloreto de metiltionínio |
| Metronidazole | Metronidazole, Metronidazole benzoate, Metronidazole hydrochloride, Metronidazole sodium |
| Minocycline | Cloridrato de minociclina, Minocycline, Minocycline hydrochloride |
| Moxifloxacin | Cloridrato de moxifloxacino, Moxifloxacin, Moxifloxacin hydrochloride |
| Neomycin | Neomycin, Neomycin sulfate |
| Nitrofurantoin | Nitrofurantoin, Nitrofurantoin\|Nitrofurantoin monohydrate, Nitrofurantoína, Nitrofurantoína_x000D_\|Nitrofurantoin monohydrate |
| Norfloxacin | Norfloxacin |
| Ofloxacin | Ofloxacin |
| Oxacillin | Oxacilina sódica, Oxacillin, Oxacillin sodium |
| Oxytetracycline | Oxytetracycline |
| Phenoxymethylpenicillin | Fenoximetilpenicilina potássica, Phenoxymethylpenicillin, Phenoxymethylpenicillin potassium |
| Pipemidic acid | Pipemidic acid |
| Piperacillin | Piperacilina sódica, Piperacillin, Piperacillin sodium |
| Piperacillin\|Sulbactam | Piperacillin\|Sulbactam |
| Piperacillin\|Tazobactam | Piperacilina sódica_x000D_\|Tazobactam, Piperacilina sódica_x000D_\|Tazobactam sódico, Piperacillin sodium\|Tazobactam sodium, Piperacillin_x000D_ \|Tazobactam, Piperacillin\|Tazobactam |
| Polymyxin b | Polymyxin, Polymyxin b, Polymyxin b sulfate, Sulfato de polimixina b |
| Procaine benzylpenicillin | Benzilpenicilina procaína, Procaine benzylpenicillin |
| Procaine benzylpenicillin\|Dipirona | Dipirona_x000D_\|Benzilpenicilina procaína |
| Rifampicin | Rifampicin |
| Secnidazole | Secnidazole |
| Sparfloxacin | Sparfloxacin |
| Spiramycin | Spiramycin |
| Sulbactam | Sulbactam |
| Sulfadiazine | Sulfadiazine |
| Sulfamethoxazole | Sulfamethoxazole |
| Sulfamethoxazole\|Trimethoprim | Sulfamethoxazole_x000D_\|Trimethoprim, Sulfamethoxazole\|Trimethoprim |
| Sulfamoxole\|Trimethoprim | Sulfamoxole_x000D_\|Trimethoprim |
| Sultamicillin | Sultamicilina, Sultamicillin, Sultamicillin tosilate, Sultamicillin tosilate dihydrate, Tosilato de sultamicilina |
| Tazobactam | Tazobactam |
| Tedizolid | Tedizolid phosphate |
| Teicoplanin | Teicoplanin |
| Terizidone | Terizidone |
| Tetracycline | Cloridrato de tetraciclina, Tetracycline |
| Thiamphenicol | Thiamphenicol glycinate acetylcysteine |
| Tigecycline | Tigecycline |
| Tinidazole | Tinidazole |
| Tobramycin | Sulfato de tobramicina, Tobramycin, Tobramycin sulfate |
| Trastuzumab | Trastuzumab, Trastuzumab dttb, Trastuzumab anns, Trastuzumab pkrb, Trastuzumab qyyp, Trastuzumabe, Trastuzumab biosimilar 2, Trastuzumab strf |
| Trimethoprim | Trimethoprim |
| Vancomycin | Cloridrato de vancomicina, Vancomycin, Vancomycin hydrochloride |

# Supplementary Table S3. Characteristics of systemic antibacterial (ATC J01) drug–event pairs by error classification, VigiMed (Brazil, 2018–2025).

| **Variable** | **Category** | **J01 Adverse Event**  **(non-medication error) *n* = 55,028** | **Medication Error**  **(non-dosing-related) *n* = 5,583** | **Dosing-related error**  ***n* = 2,587** |
| --- | --- | --- | --- | --- |
| **Sex** | Female | 30,312 (55.08%) | 2,742 (49.11%) | 1,331 (51.45%) |
|  | Male | 22,965 (41.73%) | 2,509 (44.94%) | 1,151 (44.49%) |
|  | Not reported / Unknown | 1,751 (3.18%) | 332 (5.95%) | 105 (4.06%) |
| **Age Group** | Neonate (0-30 days) | 485 (0.88%) | 185 (3.31%) | 107 (4.14%) |
|  | Infant (31 days - 5 years) | 3,602 (6.55%) | 450 (8.06%) | 180 (6.96%) |
|  | Child (6-12 years) | 2,487 (4.52%) | 196 (3.51%) | 73 (2.82%) |
|  | Adolescent (13-18 years) | 1,896 (3.45%) | 90 (1.61%) | 36 (1.39%) |
|  | Adult (19-64 years) | 30,235 (54.94%) | 1,754 (31.42%) | 976 (37.73%) |
|  | Older Adults (65+ years) | 12,176 (22.13%) | 1,309 (23.45%) | 741 (28.64%) |
|  | Not reported / Unknown | 4,147 (7.54%) | 1,599 (28.64%) | 474 (18.32%) |
| **Reporting source** | Healthcare services | 36,735 (66.76%) | 5,022 (89.95%) | 2,317 (89.56%) |
|  | Pharmaceutical companies / Industry | 9,374 (17.03%) | 367 (6.57%) | 192 (7.42%) |
|  | Patients and Healthcare Professionals | 6,197 (11.26%) | 150 (2.69%) | 65 (2.51%) |
|  | VigiMobile | 2,229 (4.05%) | 30 (0.54%) | 7 (0.27%) |
|  | VigiFlow eForms | 481 (0.87%) | 13 (0.23%) | 6 (0.23%) |
|  | Vaccination services | 7 (0.01%) | 1 (0.02%) | 0 (0.00%) |
|  | Not reported / Unknown | 5 (0.01%) | 0 (0.00%) | 0 (0.00%) |
| **Event seriousness** | Yes | 12,826 (23.31%) | 117 (2.10%) | 106 (4.10%) |
|  | No | 27,639 (50.21%) | 2,218 (39.73%) | 1,179 (45.57%) |
|  | Not reported / Unknown | 14,570 (26.48%) | 3,248 (58.18%) | 1,302 (50.33%) |

ATC: Anatomical Therapeutic Chemical classification.

# Supplementary Figure S1. Distribution of the five most frequently reported antibiotics, associated with dosing-related error drug–event pairs within each age group, expressed as unadjusted percentages, VigiMed (Brazil, 2018-2025; *n =* 2,587)

Abbreviations: AMK, amikacin; AMC, amoxicillin–clavulanate; AMP, ampicillin; AMS, amoxicillin; AZM, azithromycin; CIP, ciprofloxacin; CRO, ceftriaxone; FEP, cefepime; GEN, gentamicin; MEM, meropenem; MTZ, metronidazole; OXA, oxacillin; PEN-G, benzylpenicillin; TZP, piperacillin–tazobactam; VAN, vancomycin.

# Supplementary Table S4. Frequency of antibiotics associated with dosing-related error drug–event pairs in VigiMed (Brazil, 2018–2025, *n* = 2,587)

| **Antibiotic** | ***n*** |
| --- | --- |
| Ceftriaxone | 269 |
| Piperacillin\|Tazobactam | 258 |
| Vancomycin | 234 |
| Meropenem | 233 |
| Amikacin | 124 |
| Ciprofloxacin | 117 |
| Clindamycin | 110 |
| Amoxicillin\|Clavulanic acid | 94 |
| Azithromycin | 91 |
| Cefazolin | 90 |
| Oxacillin | 87 |
| Cefepime | 78 |
| Gentamicin | 74 |
| Metronidazole | 69 |
| Ampicillin\|Sulbactam | 58 |
| Ampicillin | 57 |
| Polymyxin b | 54 |
| Linezolid | 41 |
| Cefalexin | 38 |
| Amoxicillin | 34 |
| Avibactam\|Ceftazidime | 27 |
| Levofloxacin | 27 |
| Sulfamethoxazole\|Trimethoprim | 27 |
| Teicoplanin | 27 |
| Cefuroxime | 24 |
| Tigecycline | 23 |
| Benzylpenicillin | 22 |
| Daptomycin | 18 |
| Ertapenem | 18 |
| Piperacillin | 18 |
| Ceftazidime | 15 |
| Clarithromycin | 15 |
| Cefalotin | 12 |
| Benzathine benzylpenicillin | 11 |
| Cilastatin\|Imipenem | 9 |
| Erythromycin | 9 |
| Nitrofurantoin | 9 |
| Ceftaroline | 6 |
| Ceftolozane\|Tazobactam | 6 |
| Doxycycline | 6 |
| Fosfomycin | 6 |
| Moxifloxacin | 6 |
| Tazobactam | 4 |
| Tobramycin | 4 |
| Cefotaxime | 3 |
| Norfloxacin | 3 |
| Sultamicillin | 3 |
| Amoxicillin\|Sulbactam | 2 |
| Aztreonam | 2 |
| Cefadroxil | 2 |
| Cefpodoxime proxetil | 2 |
| Erythromycin\|Sulfafurazole | 2 |
| Benzathine benzylpenicillin\|Benzylpenicillin\|Procaine benzylpenicillin | 1 |
| Benzilpenicilina benzatina\|Benzylpenicillin | 1 |
| Benzylpenicillin\|Procaine benzylpenicillin | 1 |
| Cefdinir | 1 |
| Loracarbef | 1 |
| Procaine benzylpenicillin | 1 |
| Sulbactam | 1 |
| Sulfamethoxazole | 1 |
| Thiamphenicol | 1 |

Supplementary Table S5. Crude and adjusted prevalence ratios for medication-error classification among J01 drug–event pairs, VigiMed (Brazil, 2018–2025).

| **Variable** | **Category** | **Crude PR** | **Crude p-value** | **Adjusted PR** | **Adjusted p-value** |
| --- | --- | --- | --- | --- | --- |
| **Age Group** | Neonate (0-30 days) | 4.54 (4.01–5.14) | <0.001 | 3.90 (3.45–4.41) | <0.001 |
|  | Infant (31 days - 5 years) | 1.80 (1.63–1.99) | <0.001 | 1.78 (1.61–1.97) | <0.001 |
|  | Child (6-12) | 1.18 (1.01–1.37) | 0.032 | 1.16 (0.99–1.35) | 0.062 |
|  | Adolescent (13-18 years) | 0.75 (0.60–0.94) | 0.013 | 0.68 (0.54–0.86) | <0.001 |
|  | Adult (19-64) | 1.00 (Reference) |  | 1.00 (Reference) |  |
|  | Older adult (≥65) | 1.74 (1.62–1.87) | <0.001 | 1.70 (1.59–1.82) | <0.001 |
|  | Not reported / Unknown | 4.02 (3.73–4.34) | <0.001 | 4.24 (3.97–4.52) | <0.001 |
| **Sex** | Male | 1.00 (Reference) |  | 1.00 (Reference) |  |
|  | Female | 0.86 (0.82–0.91) | <0.001 | 0.93 (0.89–0.98) | 0.007 |
|  | Not reported / Unknown | 1.45 (1.29–1.63) | <0.001 | 0.60 (0.54–0.68) | <0.001 |
| **Reporting source** | Healthcare services | 1.00 (Reference) |  | 1.00 (Reference) |  |
|  | Pharmaceutical companies | 0.34 (0.30–0.38) | <0.001 | 0.30 (0.26–0.34) | <0.001 |
|  | Patients and healthcare professionals | 0.20 (0.17–0.23) | <0.001 | 0.25 (0.21–0.29) | <0.001 |
|  | VigiMobile | 0.10 (0.07–0.14) | <0.001 | 0.12 (0.08–0.18) | <0.001 |
|  | VigiFlow eForms | 0.23 (0.14–0.37) | <0.001 | 0.27 (0.16–0.43) | <0.001 |

The outcome comprised any medication error, including dosing- and non-dosing-related errors. The reference outcome group comprised drug–event pairs without medication errors. Crude PRs were estimated using univariable models, and adjusted PRs using a multivariable model including age group, sex, and reporting source. Variance was clustered at the report level.

# Supplementary Table S6. Crude and adjusted prevalence ratios for the association between AWaRe antibiotic classification and dosing-related error classification among medication-error drug–event pairs, VigiMed (Brazil, 2018–2025).

| **Variable** | **Category** | **Crude PR**  **(95% CI)** | **Crude**  **p-value** | **Adjusted PR**  **(95% CI)** | **Adjusted**  **p-value** |
| --- | --- | --- | --- | --- | --- |
| **AWaRe class** | Access (reference) | Reference |  | Reference |  |
|  | Watch | 1.15 (1.08–1.23) | <0.001 | 1.14 (1.07–1.22) | <0.001 |
|  | Reserve | 1.38 (1.22–1.57) | <0.001 | 1.31 (1.16–1.49) | <0.001 |
| **Age group** | Neonate (0–30 days) | 1.01 (0.87–1.18) | 0.888 | 1.08 (0.92–1.27) | 0.340 |
|  | Infant (31 days–5 years) | 0.80 (0.70–0.91) | <0.001 | 0.82 (0.71–0.94) | 0.003 |
|  | Child (6–12) | 0.76 (0.62–0.93) | 0.008 | 0.78 (0.64–0.95) | 0.016 |
|  | Adolescent (13–18) | 0.80 (0.59–1.07) | 0.134 | 0.83 (0.62–1.11) | 0.201 |
|  | Adult (19–64; reference) | Reference |  | Reference |  |
|  | Older adult (≥65) | 1.01 (0.93–1.10) | 0.759 | 1.00 (0.92–1.08) | 0.940 |
|  | Not reported / Unknown | 0.64 (0.58–0.71) | <0.001 | 0.65 (0.59–0.72) | <0.001 |
| **Sex** | Male (reference) | Reference |  | Reference |  |
|  | Female | 1.04 (0.97–1.11) | 0.280 | 1.06 (0.99–1.13) | 0.081 |
|  | Not reported / Unknown | 0.76 (0.64–0.91) | 0.002 | 0.93 (0.78–1.12) | 0.454 |
| **Reporting source** | Healthcare services (reference) | Reference |  | Reference |  |
|  | Pharmaceutical companies | 1.09 (0.93–1.27) | 0.299 | 1.06 (0.91–1.24) | 0.474 |
|  | Patients and healthcare professionals | 0.95 (0.76–1.17) | 0.613 | 0.88 (0.71–1.09) | 0.240 |
|  | VigiMobile | 0.60 (0.30–1.21) | 0.153 | 0.59 (0.29–1.19) | 0.141 |
|  | VigiFlow eForms | 1.00 (0.49–2.05) | 0.997 | 0.93 (0.44–1.96) | 0.851 |

Adjusted prevalence ratios (PRs) and 95% confidence intervals (CIs) were estimated using modified Poisson regression with a log link and cluster-robust variance at the report level. Models were adjusted for age group, sex, and reporting source. The reference category for AWaRe class was Access. Reports originating from vaccination services were excluded from the multivariable model because of insufficient sample size. The “Pharmaceutical companies / Industry” category includes reports from Industry eReporting (E2B and manual entry). The analysis included 8,134 in the adjusted model; 2,579 were classified as dosing-related errors.

# Supplementary Table S7. Sensitivity analysis: association between AWaRe class and dosing-related error classification among medication-error drug–event pairs restricted to a “suspect” drug–event relationship, VigiMed (Brazil, 2018–2025).

| **Variable** | **Category** | **PR (95% CI)** | **p-value** |
| --- | --- | --- | --- |
| **AWaRe class** | Access (reference) | Reference |  |
|  | Watch | 1.10 (1.01–1.19) | 0.034 |
|  | Reserve | 1.23 (1.05–1.44) | 0.010 |
| **Age group** | Neonate (0–30 days) | 1.22 (1.00–1.48) | 0.050 |
|  | Infant (31 days–5 years) | 0.88 (0.75–1.04) | 0.129 |
|  | Child (6–12) | 0.81 (0.63–1.04) | 0.091 |
|  | Adolescent (13–18) | 0.93 (0.66–1.29) | 0.651 |
|  | Adult (19–64; reference) | Reference |  |
|  | Older adult (≥65) | 0.99 (0.89–1.10) | 0.907 |
|  | Not reported / Unknown | 0.80 (0.71–0.90) | <0.001 |
| **Sex** | Male (reference) | Reference |  |
|  | Female | 1.10 (1.01–1.19) | 0.030 |
|  | Not reported / Unknown | 0.89 (0.73–1.08) | 0.231 |
| **Reporting source** | Healthcare services (reference) | Reference |  |
|  | Pharmaceutical companies | 1.16 (0.99–1.35) | 0.069 |
|  | Patients and healthcare professionals | 0.95 (0.76–1.18) | 0.643 |
|  | VigiMobile | 0.63 (0.31–1.28) | 0.203 |
|  | VigiFlow eForms | 1.03 (0.49–2.13) | 0.946 |

Sensitivity analysis restricted to medication-error drug–event pairs in which the antibiotic was reported with a “suspect” drug–event relationship (n=5,927). Adjusted prevalence ratios (PRs) and 95% confidence intervals (CIs) were estimated using modified Poisson regression with a log link and cluster-robust variance at the report level. Models were adjusted for age group, sex, and reporting source. Reference categories were Access (AWaRe class), Adult (19–64 years) (age group), Male (sex), and Healthcare services (reporting source).

# Supplementary Table S8. Sensitivity analysis: adjusted prevalence ratios for the association between AWaRe classification and dosing-related error classification among medication-error drug–event pairs, additionally adjusted for calendar year of entry into the system, VigiMed (Brazil, 2018–2025).

| **Variable** | **Category** | **PR (95% CI)** | **p-value** |
| --- | --- | --- | --- |
| **AWaRe class** | Access (reference) | Reference |  |
|  | Watch | 1.11 (1.04–1.19) | 0.002 |
|  | Reserve | 1.26 (1.12–1.43) | <0.001 |
| **Age group** | Neonate (0–30 days) | 1.04 (0.89–1.22) | 0.607 |
|  | Infant (31 days–5 years) | 0.83 (0.73–0.95) | 0.007 |
|  | Child (6–12) | 0.79 (0.65–0.97) | 0.025 |
|  | Adolescent (13–18) | 0.83 (0.62–1.10) | 0.202 |
|  | Adult (19–64; reference) | Reference |  |
|  | Older adult (≥65) | 1.00 (0.92–1.08) | 0.969 |
|  | Not reported / Unknown | 0.63 (0.57–0.70) | <0.001 |
| **Sex** | Male (reference) | Reference |  |
|  | Female | 1.07 (1.00–1.14) | 0.051 |
|  | Not reported / Unknown | 0.88 (0.74–1.06) | 0.177 |
| **Reporting source** | Healthcare services (reference) | Reference |  |
|  | Pharmaceutical companies | 1.02 (0.87–1.19) | 0.792 |
|  | Patients and healthcare professionals | 0.82 (0.66–1.02) | 0.070 |
|  | VigiMobile | 0.61 (0.30–1.24) | 0.172 |
|  | VigiFlow eForms | 1.14 (0.54–2.40) | 0.735 |
| **Year of entry** | 2019 (reference) | Reference |  |
|  | 2020 | 1.19 (0.92–1.53) | 0.177 |
|  | 2021 | 1.03 (0.81–1.32) | 0.797 |
|  | 2022 | 1.00 (0.78–1.27) | 0.981 |
|  | 2023 | 0.93 (0.73–1.18) | 0.536 |
|  | 2024 | 0.74 (0.58–0.94) | 0.013 |
|  | 2025 | 0.88 (0.69–1.13) | 0.319 |

Adjusted prevalence ratios (PRs) and 95% confidence intervals (CIs) were estimated using modified Poisson regression with a log link and cluster-robust variance at the report level. The model was adjusted for age group, sex, reporting source, and year of entry. Reference categories were Access (AWaRe class), Adult (19–64 years) (age group), Male (sex), Healthcare services (reporting source), and 2019 (year of entry). Data for 2025 are partial and include reports entered up to November 2025.

# Supplementary Figure S2. Medication-error drug–event pairs by year, and proportion of dosing-related errors within each AWaRe class (VigiMed, Brazil)

Total dosing-related error drug–event pairs by year (upper panel) and proportion of dosing-related error pairs within each AWaRe class, by year (lower panel). Proportions were calculated among Access, Watch and Reserve pairs. No eligible pairs were recorded in December 2018, when VigiMed was implemented; 2025 includes data up to November and is therefore partial.

# Supplementary Table S9. STROBE checklist for cross-sectional studies.

| **Item No** | **Recommendation** | **Reported on (section; page/line)** |
| --- | --- | --- |
| **Title and abstract** | | |
| **1** | (a) Indicate the study's design with a commonly used term in the title or the abstract  (b) Provide in the abstract an informative and balanced summary of what was done and what was found | (a) Abstract: Methods (“exploratory cross-sectional analysis”); Note: the design term appears in the abstract.  (b) Structured Abstract (Background/Methods/Results/Conclusion). |
| **Introduction** | | |
| **2** | Background/rationale: explain the scientific background and rationale for the investigation | Introduction, paragraphs 1–4. |
| **3** | Objectives: state specific objectives, including any prespecified hypotheses | Introduction, final paragraph (“This study aimed to characterise…”). |
| **Methods** | | |
| **4** | Study design: present key elements of study design early in the paper | Methods 2.1 Study Design and Setting. |
| **5** | Setting: describe the setting, locations, and relevant dates, including periods of recruitment, exposure, follow-up, and data collection | Methods 2.1 (VigiMed/Anvisa; Dec 2018–Nov 2025; data retrieved 3 Dec 2025). |
| **6** | (a) Give the eligibility criteria, and the sources and methods of selection of participants | Methods 2.2 Data Processing and 2.3 Analytical Selection (ATC J01; “suspected”/“not administered”; ≥1 antibiotic + 1 AE; foetuses excluded; deduplication); Supplementary Methods S1–S3. |
| **7** | Variables: clearly define all outcomes, exposures, predictors, potential confounders, and effect modifiers. Give diagnostic criteria, if applicable | Methods 2.3 (outcome: DE binary; exposure: AWaRe class) and 2.4 (confounders: age group, sex, reporting source). |
| **8** | Data sources/measurement: for each variable of interest, give sources of data and details of methods of assessment | Methods 2.1–2.3 (WHODrug; MedDRA SMQ Medication Error v28.1; ATC J01; 2023 WHO AWaRe); Supplementary Methods S1–S3. |
| **9** | Bias: describe any efforts to address potential sources of bias | Methods 2.3–2.4 and Discussion 4.1. Addresses: inclusion of “medicine not administered” pairs and suspect-only sensitivity analysis; exclusion of seriousness on collider-bias grounds; non-random missingness retained as a distinct category; under-reporting/reporting bias. |
| **10** | Study size: explain how the study size was arrived at | Methods 2.3 — census of all eligible J01 drug–event pairs in the period. |
| **11** | Quantitative variables: explain how quantitative variables were handled in the analyses; describe groupings and why | Methods 2.3–2.4 (age grouped; AWaRe categories; calendar year as categorical covariate); Supplementary Methods S2. |
| **12** | (a) Describe all statistical methods, including those used to control for confounding  (b) Describe any methods used to examine subgroups and interactions  (c) Explain how missing data were addressed  (d) If applicable, describe analytical methods taking account of sampling strategy  (e) Describe any sensitivity analyses | (a) Modified Poisson regression, log link, PRs with 95% CI, cluster-robust variance at report level; crude and adjusted (age, sex, reporting source).  (b) Subgroups examined descriptively only: dosing-related errors grouped into timing-related and dosaging-related error domains (Table 1), and a heatmap of DE proportion by antibiotic × age group (Figure 3); no formal subgroup regression or interaction testing was performed.  (c) Not imputed (likely non-random missingness); retained as “Not reported/Unknown” category.  (d) Not applicable (spontaneous reports, no sampling design); report-level clustering handled via robust variance.  (e) Suspect-only restriction and adjustment for calendar year of system entry. |
| **Results** | | |
| **13** | (a) Report numbers of individuals at each stage of study  (b) Give reasons for non-participation at each stage  (c) Consider use of a flow diagram | (a) Results and Figure 1.  (b) Figure 1 (reasons and N for each exclusion: duplicates, foetus-related, non-J01, non-error, non-dosing).  (c) Figure 1 (study flow diagram). |
| **14** | (a) Give characteristics of study participants and information on exposures and potential confounders  (b) Indicate number of participants with missing data for each variable of interest | (a) Results and Supplementary Table S3.  (b) Missing values retained as a distinct "Not reported/Unknown" category and reported for each variable across the descriptive (Supplementary Table S3), and retained as a distinct category in the regression models (Supplementary Tables S5–S8). |
| **15** | Outcome data: report numbers of outcome events or summary measures | Results (MEPs = 8,170; DEPs n = 2,587; Table 1 by error category; distribution by AWaRe). |
| **16** | (a) Give unadjusted and, if applicable, confounder-adjusted estimates and their precision (e.g., 95% CI); make clear which confounders were adjusted for and why  (b) Report category boundaries when continuous variables were categorized  (c) If relevant, consider translating estimates of relative risk into absolute risk | (a) Table 2 and Figure 2 (adjusted PRs, 95% CI); crude PRs in Supplementary Tables S5–S6; confounders (age, sex, reporting source) specified in the Figure 2 and Table 2 legends.  (b) Age (the only categorised continuous variable) grouped using boundaries defined in Methods 2.2 and Supplementary Methods.  (c) Not applicable — spontaneous reports lack population denominators; stated in Methods 2.4 and Discussion. |
| **17** | Other analyses: report other analyses done (subgroups, interactions, sensitivity analyses) | Contextual analysis, Figure 2; heatmap, Figure 3; sensitivity analyses, Supplementary Tables S7–S8. |
| **Discussion** | | |
| **18** | Key results: summarise key results with reference to study objectives | Discussion, opening paragraph. |
| **19** | Limitations: discuss limitations, taking into account sources of potential bias or imprecision; discuss both direction and magnitude of any potential bias | Discussion and 4.1 Limitations and Strengths. |
| **20** | Interpretation: give a cautious overall interpretation considering objectives, limitations, multiplicity of analyses, results from similar studies, and other relevant evidence | Discussion (non-causal interpretation; comparison with VigiBase/LMIC and Russian data). |
| **21** | Generalisability: discuss the generalisability (external validity) of the study results | Discussion 4.1 and Conclusion (“Brazil and similar settings”). |
| **Other information** | | |
| **22** | Funding: give the source of funding and the role of the funders for the present study | Funding statement (Faperj E-26/201.373/2022; no role of sponsor). |

STROBE, Strengthening the Reporting of Observational Studies in Epidemiology; ATC, Anatomical Therapeutic Chemical classification; AWaRe, Access/Watch/Reserve; CI, confidence interval; DEP, dosing-related error drug–event pair; MEP, medication-error drug–event pair; MedDRA, Medical Dictionary for Regulatory Activities; PR, prevalence ratio; PT, Preferred Term; SMQ, Standardised MedDRA Query. Checklist obtained from www.strobe-statement.org.
