# Supplementary material for: Reported Dosing‐Related Medication Errors Involving Systemic Antibiotics in the Brazilian Pharmacovigilance Database (2018–2025): Distribution Across the WHO AWaRe Classification
Source: Pharmacoepidemiol Drug Saf. 2026 Oct 7;35(10):e70496. doi: 10.1002/pds.70496 (PMC13643816; doi:10.1002/pds.70496)
Supplement: Supplementary file 2 [file PDS-35-e70496-s002.docx]

Supplementary Methods

[**Supplementary Methods** **S1. Detailed Deduplication Algorithm**	2](#_Toc223072520)

[**Supplementary Methods** **S2. Variable Harmonisation**	2](#_Toc223072521)

[**Supplementary Methods** **S3. Detailed Analytical Selection Criteria**	3](#_Toc223072522)

# **Supplementary Methods** **S1. Detailed Deduplication Algorithm**

A deterministic record-linkage deduplication strategy was performed hierarchically in two sequential levels. In the first level, only reports with complete information on medicine (NOME_MEDICAMENTO_WHODRUG), event (PT), sex (SEXO), date of birth (DATA_NASCIMENTO), and event onset date (DATA_INICIO_HORA) were eligible for deduplication, using a composite deterministic key that included the temporal marker. Reports with any missing element of this key were excluded from this step. In the second level, deduplication was applied exclusively to the remaining reports that had complete information on medicine, event, sex, and date of birth but lacked a valid onset date, using a reduced composite key. Reports not eligible for deterministic linkage were retained; however, exact duplicate rows were removed to mitigate duplication introduced by data handling or extraction steps.

This strategy was specifically adapted for large-scale Brazilian pharmacovigilance data. Due to the heterogeneity and incompleteness (Supplementary Table S1) typical of spontaneous reporting systems, the procedure was designed to prioritise reproducibility and minimise false-positive merges.

The CSV file corresponding to the reports (VigiMed_Notificacoes.csv), which contains the necessary identifiers for deduplication, was used as the basis for the duplicate detection workflow. Medicine and reaction terms were normalised using accent removal, lowercasing, and light punctuation stripping to minimise spelling-related variability while preserving semantic distinctions. During date cleaning, values corresponding to years before 1910 or dates later than the last data update (30 November 2025) were recoded as missing.

Reports with an identical composite key were considered duplicate candidates. Within each set of duplicates, the first record for each deduplication key was retained as the representative. Selection at the report level did not prioritise completeness of non-key fields. All members of each duplicate group were stored with group identifiers to support manual auditing.

Separately, before their respective merges, the medicines (VigiMed_Medicamentos.csv) and reactions (VigiMed_Reacoes.csv) datasets were internally deduplicated to remove repeated entries. Records sharing the same medicine key (IDENTIFICACAO_NOTIFICACAO, RELACAO_MEDICAMENTO_EVENTO, NOME_MEDICAMENTO_WHODRUG) or reaction key (IDENTIFICACAO_NOTIFICACAO, PT) were consolidated by retaining the row with the highest number of non-null fields. This procedure prevented artificial inflation of medication–event pairs due to these repeated entries.

For the analytical dataset, drug–event pairs were required to include a non-missing active substance and a reported AE identified by a PT. Pairs not meeting these criteria were excluded in a subsequent filtering step, as these fields represent the minimum information required for the analytical objectives of this study. Pairs from reports with the age group recorded as “foetus” were also excluded.

# **Supplementary Methods** **S2. Variable Harmonisation**

Age: To maximise data completeness, patient age was calculated using the date of birth and the adverse event onset date or, when unavailable, the therapy start date. When the date of birth was missing, the age at the time of the reaction as reported by the reporter was used. All age values were subsequently categorised into standardized WHO (World Health Organization) age groups: neonate (0–30 days), infant (31 days to 5 years), child (6–12 years), adolescent (13–18 years), adult (19–64 years), and elderly (≥65 years) ^35^. For records that remained missing after these steps, the age group classification directly reported in the notification was retained. This hierarchical strategy increased age group completeness to 88%, compared with 73% completeness when relying solely on date of birth.

Active substances: Although the WHODrug Global Dictionary was used, some reports included variations from previous notifications, and considerable nomenclature variability was observed. Active substance names were therefore harmonised to ensure terminological consistency (see Supplementary Table S2).

# **Supplementary Methods** **S3. Detailed Analytical Selection Criteria**

The relationship between the medicine and the adverse event (AE) is defined by the notifier and may be classified as suspected, concomitant, interacting, or medicine not administered. A suspected medicine refers to a product considered by the notifier to be related to the reported event. A concomitant medicine, meanwhile, is one that the patient is using at the same time. The CSV file corresponding to the medicines was used to identify ABs classified as ‘suspected’ or ‘medicine not administered’ ^38^.

Each report of one or more suspected antibiotics (ABs) and one or more AEs was expanded to generate all possible combinations.

Systemic antibacterials were identified using ATC code J01. Products with multiple ATC classifications that did not represent systemic antibacterials were subsequently excluded based on the harmonised active-substance classification.

AEs coded as MedDRA Preferred Terms (PTs) were classified using the Standardised MedDRA Query (SMQ) Medication Error (Brazilian Portuguese version 28.1). MedDRA® the Medical Dictionary for Regulatory Activities terminology is the international medical terminology developed under the auspices of the International Council for Harmonisation of Technical Requirements for Pharmaceuticals for Human Use (ICH). The full list of PTs is not reproduced in the manuscript. Access to the MedDRA SMQ file may be obtained through an appropriate licence or requested directly from MedDRA, subject to its approval and applicable terms.

Within the SMQ Medication Error, a predefined subset of 32 PTs explicitly referring to dose amount, dose omission, dose adjustment or titration, dosing frequency or interval, duration of administration, or rate of administration was selected to define dosing-related errors. Of these 32 PTs, 23 occurred in the analytical dataset and are presented with their corresponding classification in Supplementary Methods Table 1.

Supplementary Methods Table 1. Dosing-related error terms identified in the study dataset and their classification (Brazil, VigiMed, 2018–2025).

| **Classification** | **MedDRA Preferred Term** |
| --- | --- |
| **Dose omission** | Drug dose omission by device |
|  | Product dose omission in error |
|  | Product dose omission issue |
| **Dosing regimen problem** | Incorrect product administration duration |
|  | Inappropriate schedule of product administration |
|  | Incorrect dosing regimen |
|  | Incorrect drug administration rate |
| **Incorrect dose** | Unadjusted dose administered |
|  | Incorrect dosage administered |
|  | Dosage not adjusted |
|  | Incorrect dose |
|  | Incorrect dose administered |
|  | Incorrect dose administered by device |
|  | Incorrect dose administered by product |
|  | Dose calculation error |
| **Subtherapeutic dose** | Underdose |
|  | Accidental underdose |
|  | Prescribed underdose |
| **Overdose** | Extra dose administered |
|  | Increased dose administered |
|  | Overdose |
|  | Accidental overdose |
|  | Prescribed overdose |

Preferred Terms were identified using the Brazilian Portuguese version 28.1 of MedDRA and are presented using the corresponding official English MedDRA terms.
